# From insect endosymbiont to phloem colonizer: comparative genomics unveils the lifestyle transition of phytopathogenic *Arsenophonus* strains

**DOI:** 10.1128/msystems.01496-24

**Published:** 2025-04-09

**Authors:** Mathieu Mahillon, Christophe Debonneville, Raphaël Groux, David Roquis, Justine Brodard, Franco Faoro, Xavier Foissac, Olivier Schumpp, Jessica Dittmer

**Affiliations:** 1 Research group Virology, Bacteriology and Phytoplasmology, Agroscope, Nyon, Switzerland; 2 Haute école du paysage, d'ingénierie et d'architecture de Genève 128976 , Geneva, Switzerland; 3 Dipartimento di Scienze agrarie e ambientali, Università degli Studi di Milano, Milano, Italy; 4 UMR 1332 Biologie du Fruit et Pathologie, INRAE, Université de Bordeaux, Bordeaux, France; 5 UMR 1345, Université d’Angers, Institut Agro, INRAE, IRHS, SFR Quasav, Angers, France; CNRS Delegation Alpes, Villeurbanne, France

**Keywords:** *Arsenophonus*, cixiid, endosymbiont, phloem, Phlomobacter, planthopper, proteobacteria, xylellain

## Abstract

Bacteria infecting the plant phloem represent a growing threat worldwide. While these organisms often resist *in vitro* culture, they multiply both in plant sieve elements and hemipteran vectors. Such cross-kingdom parasitic lifestyle has emerged in diverse taxa via distinct ecological routes. In the genus *Arsenophonus*, the phloem pathogens “*Candidatus* Arsenophonus phytopathogenicus” (Ap) and “*Ca*. Phlomobacter fragariae” (Pf) have evolved from insect endosymbionts, but the genetic mechanisms underlying this transition have not been explored. To fill this gap, we obtained the genomes of both strains from insect host metagenomes. The resulting assemblies are highly similar in size and functional repertoire, rich in viral sequences, and closely resemble the genomes of several facultative endosymbiotic *Arsenophonus* strains of sap-sucking hemipterans. However, a phylogenomic analysis demonstrated distinct origins, as Ap belongs to the “*Triatominarum*” clade, whereas Pf represents a distant species. We identified a set of orthologs encoded only by Ap and Pf in the genus, including hydrolytic enzymes likely targeting plant substrates. In particular, both bacteria encode putative plant cell wall-degrading enzymes and cysteine peptidases related to xylellain, a papain-like peptidase from *Xylella fastidiosa*, for which close homologs are found in diverse *Pseudomonadota* infecting the plant vasculature. *In silico* predictions and gene expression analyses further support a role during phloem colonization for several of the shared orthologs. We conclude that the double emergence of phytopathogenicity in *Arsenophonus* may have been mediated by a few horizontal gene transfer events, involving genes acquired from other *Pseudomonadota*, including phytopathogens.

**IMPORTANCE:**

We investigate the genetic mechanisms of a transition in bacterial lifestyle. We focus on two phloem pathogens belonging to the genus *Arsenophonus*: “*Candidatus* Arsenophonus phytopathogenicus” and “*Ca*. Phlomobacter fragariae.” Both bacteria cause economically significant pathologies, and they have likely emerged among facultative insect endosymbionts. Our genomic analyses show that both strains are highly similar to other strains of the genus associated with sap-sucking hemipterans, suggesting a recent lifestyle shift. Importantly, although the phytopathogenic *Arsenophonus* strains belong to distant clades, they share a small set of orthologs unique in the genus pangenome. We provide evidence that several of these genes produce hydrolytic enzymes that are secreted and may target plant substrates. The acquisition and exchange of these genes may thus have played a pivotal role in the lifestyle transition of the phytopathogenic *Arsenophonus* strains.

## INTRODUCTION

In vascular plants, the phloem ensures the translocation of photosynthates from source to sink tissues. As a sugar-rich environment, the phloem also represents a niche for pathogenic microorganisms (1, 2). In particular, bacterial infections restricted to the sieve elements (SEs; i.e., the phloem conductive cells) are difficult to control and cause damaging diseases worldwide (3). The causal agents are generally non-culturable and “vector-borne” as they have evolved a biphasic lifestyle, alternating between plant SEs and sap-sucking hemipteran vectors (4). Notorious examples of these bacteria include *Candidatus* (*Ca*.) species of the genera *Phytoplasma* and *Liberibacter*. This lifestyle is shared by bacteria from distant taxa, and it can emerge through distinct evolutionary routes (3, 5). In the phylum *Pseudomonadota* (formerly *Proteobacteria*), vector-borne phloem pathogens have emerged either among plant-associated bacteria as in the genera *Liberibacter* and *Serratia* (1, 6, 7), or among insect endosymbionts as proposed for the genera *Rickettsia* and *Arsenophonus* (5, 8). In the latter, two phytopathogens have been identified, namely, “*Ca*. Arsenophonus phytopathogenicus” (Ap) and “*Ca*. Phlomobacter fragariae” (Pf) (9–11).

Ap is transmitted by the planthoppers *Pentastiridius leporinus* and *Cixius wagneri*, and it also colonizes the SEs of sugar beet, thereby inducing the syndrome “basses richesses” (SBR). SBR was first described in France and now occurs in Germany and Switzerland as well (12–14). Recently, Ap has been detected in sugar beets across Central Europe as well as in potato and onion plants in Germany and Switzerland (15–19), indicating its growing distribution and host range and warning of potential novel epidemics. In contrast, Pf is transmitted by *C. wagneri* and causes strawberry marginal chlorosis (SMC) (20), first reported in France (21) and then in Japan (22). SMC was also documented in Italy, where it was associated with both Pf and Ap (23, 24).

While Ap and Pf are phytopathogens, other *Arsenophonus* strains are known as arthropod endosymbionts. *Arsenophonus* belongs to the family *Morganellaceae* in the order *Enterobacterales* (25). It represents one of the most widespread insect endosymbiotic genera (26), and its host range also comprises other arthropod groups such as arachnids (27). Host-symbiont interactions, tissue tropisms, and transmission routes of *Arsenophonus* strains are diverse (28, 29). Infections can be associated with reproductive parasitism (30), insecticide resistance (31), feeding behavior alteration (32, 33), and nutritional symbiosis (34). These bacteria can multiply extra- or intracellularly, and some are maternally inherited. The best-studied species is *Arsenophonus nasoniae*, an endosymbiont of parasitoid wasps sometimes associated with “male-killing” (35). Transmission of *A. nasoniae* can be vertical but also horizontal through multi-parasitism events (36). Horizontal transmission has also been documented for the bee endosymbiont *Arsenophonus apicola* (37). Unlike these two species, many members of the genus can only be grown in host cells or even completely resist *in vitro* culture (5). Some strains have become obligate “primary” endosymbionts (P-endosymbionts) (38, 39). This great lifestyle diversity is mirrored by various genomic architectures. While the complex genome of *A. nasoniae* consists of one large circular chromosome (>3.5 Mb) and 7–20 extrachromosomal elements (40, 41), the genomes of P-endosymbionts are highly eroded (<1.2 Mb), lack extrachromosomal elements, and display reduced metabolisms streamlined for the production of nutrients lacking from the host’s diet (38, 39).

Several aspects support the idea of an ancestral insect-restricted lifestyle and the recent emergence of phytopathogenicity in *Arsenophonus*. (i) All known strains of the genus (including the phytopathogenic ones) are associated with arthropods. (ii) Some *Arsenophonus* strains are P-endosymbionts of insects with highly reduced genomes, indicating millions of years of co-evolution with certain insect species. In contrast, there is no evidence for similar long-lasting relationships between *Arsenophonus* and plants. (iii) The genetic diversity among phytopathogenic strains is very low, suggesting a recent emergence (10). (iv) Ap and Pf conserve traits typically associated with insect endosymbionts (e.g., high infection rate, vertical transmission, and presence in reproductive organs) (11). Furthermore, phylogenetic and ancestral state reconstruction analyses support this scenario (11). Accordingly, Ap and Pf represent ideal models to investigate the genetic changes involved in the transition from insect endosymbiont to vector-borne phloem pathogen. Probable mechanisms underlying this transition would be the acquisition of new functions, such as virulence factors, through horizontal gene transfer (HGT). To investigate this possibility, we compiled the first genome assemblies for Pf and Ap. As both bacteria are currently non-culturable, we produced hybrid assemblies for Pf (Pf-FR) and Ap (Ap-CH) using metagenomes of insects recently collected in France and Switzerland, respectively. In addition, the assembly of a second strain of Ap (Ap-FR) was obtained using short reads generated from insects sampled during an earlier outbreak in France. Following a genus-wide comparative analysis, we identified orthologs exclusively shared by the phytopathogenic strains that may have enabled their lifestyle transition.

## RESULTS

### General features of the assemblies

**Fig 1:**
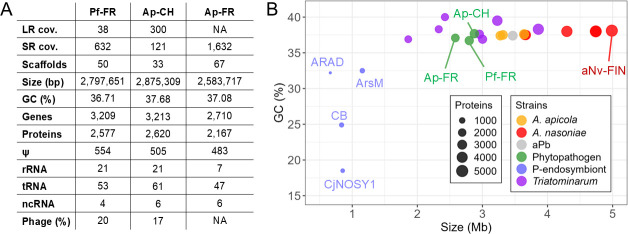
(**A**) Global genomic features of Pf-FR and Ap-CH/FR. LR: long reads; SR: short reads. (**B**) Distribution of GC content (in percent) and size (in megabases [Mb]) for the *Arsenophonus* genomes. Each point corresponds to a single genome. Strain names are provided for the largest and smallest genomes as well as the phytopathogens. Details on host species, transmission mode, and phenotype of each strain are provided in Table 1.

The genome sizes of Ap and Pf (2.6–2.9 Mb) are much larger than those reported for other vector-borne phloem pathogens such as liberibacters, phytoplasmas, and spiroplasmas (<1.7 Mb [42]). The main features of the three assemblies are summarized in Fig. 1A. The hybrid assembly approach produced 50 and 33 scaffolds for Pf-FR and Ap-CH, respectively. The best assembly was obtained for the latter, as 28% of the genome is contained in the longest scaffold (806 kb) and the genome N50 is 384 kb (97 kb for Pf-FR). The genome of Ap-FR is shorter and more discontinuous with 67 scaffolds and an N50 of 106 kb. The three assemblies exhibit a guanine-cytosine (GC) content of ca. 37%, which is similar to other facultative endosymbiotic *Arsenophonus* strains, whereas the genomes of P-endosymbionts are richer in adenine-thymine (AT) (Fig. 1B). The genome sizes, numbers of predicted proteins (2,100–2,500), and pseudogenization rates (15.7%–17.8 %) of the assemblies occupy an intermediate position within the genus and align with several strains of the *Triatominarum* clade (Fig. 1B) (43).

CheckM analyses confirmed a high level of genome completeness for the three assemblies: 96.76% for Ap-FR, 97.84% for Pf-FR, and 99.46% for Ap-CH. In the latter, seven complete rRNA operons were identified, while a previous study found only six copies of the 16S rRNA (44). Three complete rRNA operons were detected for Pf-FR, but this genome likely contains at least seven rRNA operons as additional rRNA genes occur at scaffold ends. Both Ap genomes show extensive similarities (Fig. S1); hence, some of the finer-scale analyses presented herein will focus only on Ap-CH.

### Phage and plasmid regions

Despite high sequencing coverages and multiple assembly approaches, the three assemblies could not be closed, consistent with previous *Arsenophonus* genome projects necessitating DNA from pure cultures (41, 45). The assemblies of Ap and Pf may thus contain extrachromosomal elements, as reported for other strains of the genus (Table 1) (43). Several *Arsenophonus* extrachromosomal elements were recently characterized as “phage-plasmids,” harboring both phage and plasmid modules (41, 46), and some correspond to helper prophages and their associated satellites (47).

**TABLE 1:** Genomes of *Arsenophonus* strains used in this study

Strain	Host species	Transmission mode^*a*^	Phenotype	Genome size (Mb)	Assembly status^*b*^	Plasmid number	Accession	Reference
Vector-borne phytopathogens								
*Ca*. Ap-CH	*P. leporinus*	M	Plant pathogen	2.9	S	NA	GCA_047291415.1	This study
*Ca*. Ap-FR	*P. leporinus*	M	Plant pathogen	2.6	S	NA	GCA_047291325.1	This study
*Ca*. Pf-FR	*C. wagneri*	H	Plant pathogen	2.8	S	NA	GCA_047291315.1	This study
Facultative endosymbionts								
*A. nasoniae* aNv-CAN	*Nasonia vitripennis*	M	Male-killing	5.1	C	20	GCF_029873515.1	(43)
*A. nasoniae* aNv-CH	*Nasonia vitripennis*	M	Male-killing	4.7	C	16	GCF_029873535.1	(37)
*A. nasoniae* aNv-DSM	*Nasonia vitripennis*	M	Male-killing	3.6	S	NA	GCF_000429565.1	(45)
*A. nasoniae* aNv-FIN	*Nasonia vitripennis*	M	Male-killing	5.0	C	17	GCF_004768525.1	(41)
*A. nasoniae* aNv-UK	*Nasonia vitripennis*	M	Male-killing	4.7	C	16	GCF_029873555.1	(43)
*A. nasoniae* aPv	*Pachycrepoideus vindemmiae*	V	Unknown	4.3	C	18	GCF_029873495.1	(43)
*A. nasoniae* aIh	*Ixodiphagus hookeri*	V	Unknown	3.7	C	7	GCF_029873455.1	(43)
*A. apicola* aApi-US	*Apis mellifera*	H	Putative pathogen	3.6	C	6	GCF_020268605.1	(48)
*A. apicola* aApi-CH	*Apis mellifera*	H	Putative pathogen	3.3	S	NA	GCF_903968575.1	(37)
*A. apicola* aApi-AU	*Apis mellifera*	H	Putative pathogen	3.2	C	5	GCF_029906405.1	(43)
*A. triatominarum* ATi	*Triatoma infestans*	V	Unknown	3.9	S	NA	GCF_001640365.1	(42)
*Arsenophonus* sp. aPb	*Polyommatus bellargus*	H	Unknown	3.5	C	4	GCF_029873475.1	(49)
*Ca*. Arsenophonus sp. ENCA	*Entylia carinata*	NA	Unknown	3.2	S	NA	GCF_002287155.1	(50)
*Ca*. Arsenophonus sp. ARAF	*Aleurodicus floccissimus*	V	Provides B vitamins	3.0	S	NA	GCF_900343025.1	(39)
*Ca*. Arsenophonus sp. Hangzhou	*Nilaparvata lugens*	V	Likely provides B vitamins	2.9	S	NA	GCF_000757905.1	(51)
*Ca*. Arsenophonus sp. Ash	*Aphis craccivora*	V	Mediates plant host range	2.4	C	1	GCF_013460135.1	–^*d*^
*Ca*. Arsenophonus sp. Asia-II-3	*Bemisia tabaci*	V	Provides B vitamins	2.3	S	NA	GCF_004118055.1	(52)
*Ca*. Arsenophonus sp. MEDQ21	*Bemisia tabaci*	V	Likely provides B vitamins	1.9	S	NA	GCF_902713415.1	–
P-endosymbionts								
*Ca*. A. melophagi ArsM	*Melophagus ovinus*	V	Provides B vitamins	1.1	S	NA	NA^*c*^	(34)
*Ca*. Arsenophonus sp. CjNOSY1	*Ceratovacuna japonica*	V	Provides riboflavin	0.9	C	0	GCA_024349725.1	(53)
*Ca*. A. lipoptenae CB	*Lipoptena cervi*	V	Provides B vitamins	0.8	C	0	GCF_001534665.1	(38)
*Ca*. Arsenophonus sp. ARAD	*Aleurodicus dispersus*	V	Provides B vitamins	0.6	C	0	GCF_900343015.1	(39)

^*a*^ H: horizontal; V: vertical; M: mixed.

^*b*^ S: scaffolds; C: closed.

^*c*^ Genome available online: http://users.prf.jcu.cz/novake01.

^*d*^ –, no reference available for this genome.

**Fig 2:**
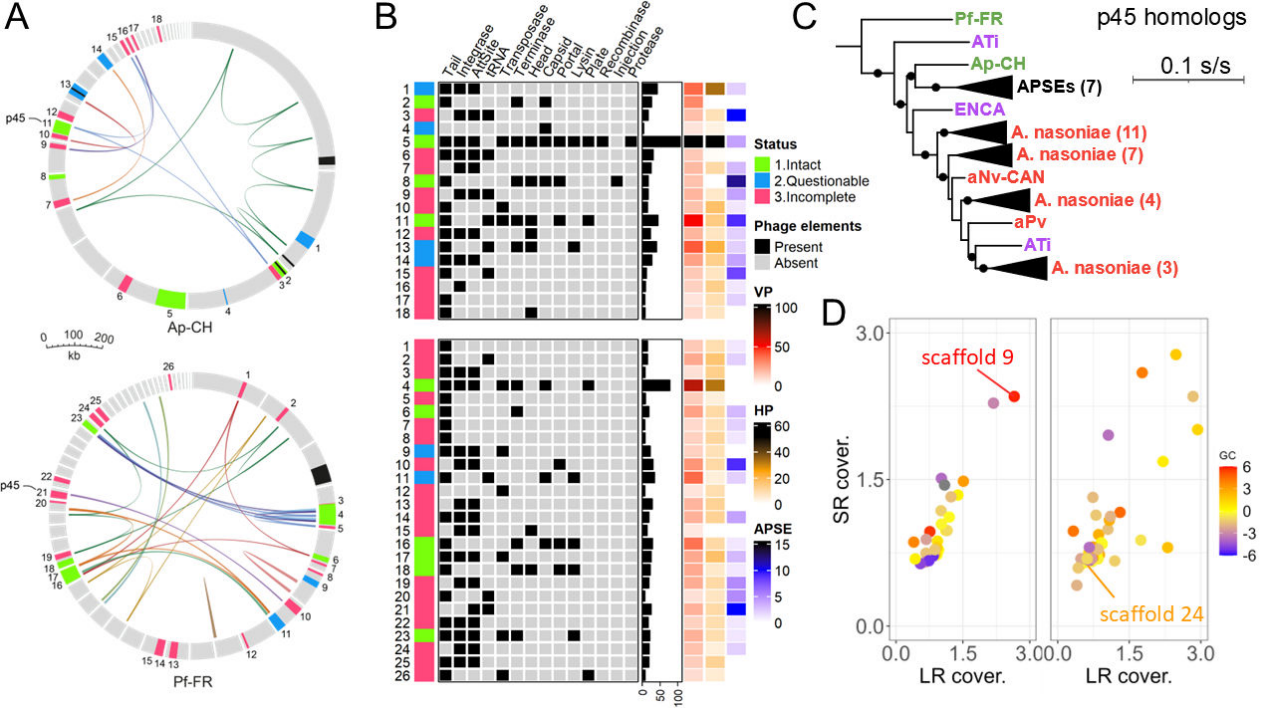
Viral content in Ap-CH and Pf-FR. (**A**) Schematic representation of the genome assemblies of Ap-CH (top) and Pf-FR (bottom). Each assembly is represented as a circle accommodating the scaffolds. Black blocks indicate regions syntenic to *Arsenophonus* extrachromosomal elements. Other colored blocks represent phage regions (>1 kb) identified by Phaster. Inner ribbons connect syntenic blocks (>5 kb). (**B**) Composition of the phage regions. VP: number of viral proteins; HP: number of hypothetical non-viral proteins; APSE: number of APSE proteins. The barplot represents the size of each region in kb. (**C**) Maximum-likelihood phylogenetic tree of the p45 homologs, built with the model JTT + F + I + G4. Black circles on branches indicate >50% bootstrap support. The scale is given in substitutions per site. Strains are color-coded as in Fig. 1B. Polymerases from *Citrobacter koseri* (WP_200053203.1) and *Salmonella enterica* (EFS9905187.1) were used to root the initial tree. (**D**) Normalized long-read (LR) and short-read (SR) coverages for the scaffolds of Ap-CH (left) and Pf-FR (right). The color scale indicates the difference in GC content from the assembly average.

Four regions in Ap-CH and one in Pf-FR exhibit synteny with sequences from known *Arsenophonus* extrachromosomal elements (Fig. 2A, black blocks, and Table S2). Plasmid genes are present both inside and outside of these regions in both assemblies (Table S3). Phaster predicted 18 and 26 phage regions for Ap-CH and Pf-FR, respectively, representing 17%–20% of the genomes (Fig. 2A, green, blue, and red blocks). These repetitive regions likely contributed to the assembly breaks, as indicated by their locations at scaffold ends (Fig. 2A, inner ribbons). Most of these regions are short and predicted as “incomplete,” harboring only a few viral genes (Fig. 2B). Their status should thus be taken with caution since they may represent phage relics or partial sequences. Conversely, eight regions for each strain are classified as “questionable” and even “intact” and could represent functional phages.

Importantly, one intact phage DNA polymerase was detected in both hybrid assemblies (ApCH_3206 and PfFR_1270). They represent homologs of p45 (>87% aa id.), the polymerase of *Acyrthosiphon pisum* secondary endosymbiont (APSE) phages that infect the pea aphid endosymbiont *Hamiltonella defensa* (*Gammaproteobacteria*) (54). Similar p45 homologs are found in other *Arsenophonus* strains (Fig. 2C) which also harbor APSE-like phages that can be part of modules forming mosaics with unrelated phages (54). Likewise, in Ap-CH and Pf-FR, numerous APSE genes are found throughout the assemblies (Fig. 2B, column “APSE”), and the p45 homologs are located within clusters syntenic to APSE replicative modules 1 and 2 (Fig. S2). In the case of Ap-CH, these modules are part of the intact phage region 11 on scaffold 9, which also contains the incomplete phage region 10 and several plasmid genes (Table S3). This scaffold exhibits a high GC content of 43% and a high sequencing coverage (Fig. 2D), indicating that it may be a multi-copy phage-plasmid element. In Pf-FR, the APSE replicative modules are located within the incomplete phage region 21 on scaffold 24, which shows no discrepancy in coverage and GC content compared to other scaffolds (Fig. 2D), and might hence represent a single-copy region. These modules could be associated with an integrated prophage since the rest of the scaffold consists of non-viral genes. Unfortunately, the APSE replicative modules of both assemblies are located at scaffold ends (Fig. S2), limiting further characterization.

### Phylogenomic relationships

**Fig 3:**
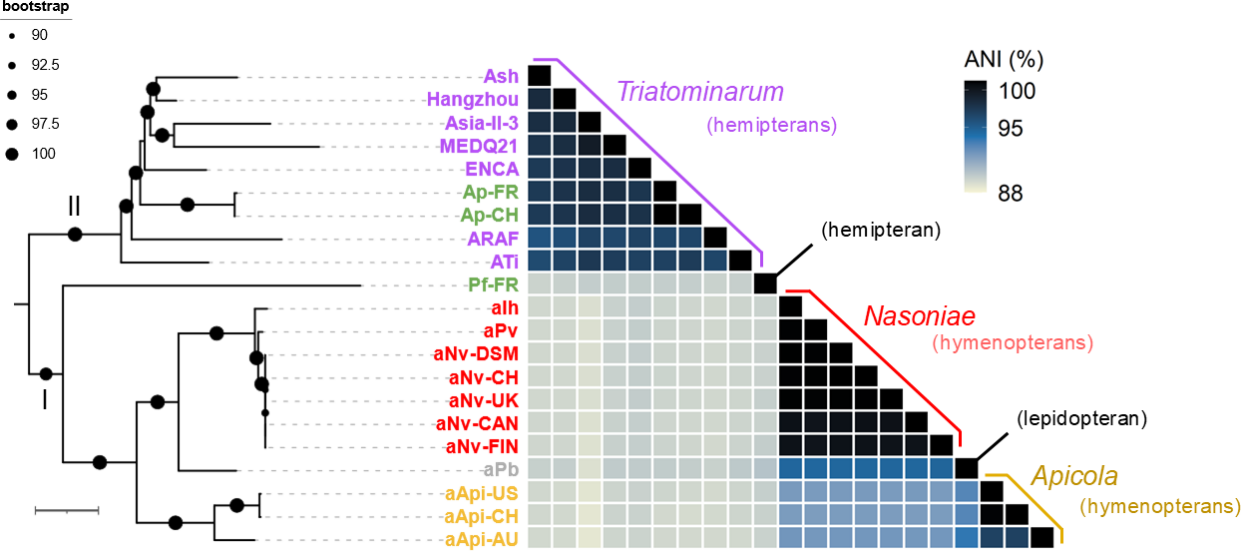
Phylogenomic and taxonomic analyses of facultative endosymbiotic *Arsenophonus* strains. Left: ML phylogenomic tree based on 118 single copy protein-coding genes. All branches have >90% bootstrap support. Genes from *P. stuartii* and *P. mirabilis* were used to root the initial tree. The tree scale represents 0.01 substitution per site. Basal clades are indicated by Latin numbers. Right: matrix of pairwise average nucleotide identity (ANI; in percent). Strains are color-coded as in Fig. 1B. The host ranges are given in parentheses.

Both maximum-likelihood and Bayesian phylogenomic analyses combining Ap, Pf, and other facultative *Arsenophonus* symbionts produced congruent tree topologies (Fig. 3; Fig. S3, respectively). Specifically, they revealed two basal clades within the genus (“I” and “II”), consistent with previous studies (48, 49). Clade I accommodates the hymenoptera-infecting parasitic species *Nasoniae* and *Apicola* as well as the butterfly-infecting strain aPb. Clade II, or “*Triatominarum*” clade (43), comprises hemipteran endosymbionts, some of which (strains ARAF, Asia-II-3, Hangzhou) are vertically transmitted and likely provide their hosts with B vitamins, similar to P-endosymbionts (Table 1) (39, 51, 52).

In both trees, Ap and Pf unambiguously represent distinct species. The Ap strains are deeply embedded within the *Triatominarum* clade (bootstrap support: 100%, posterior probability: 1), whose members can be considered as strains of the same “species complex” since they share very high ANI levels (>95% [55]). In contrast, Pf is an early-branching member of clade I (bootstrap support: 98%, posterior probability: 1) and shares low ANI levels (<91%) with the other strains, indicating that it represents a distinct species within the genus. Additional analyses including the P-endosymbionts produced similar tree topologies, albeit with reduced branch support due to long-branch attractions caused by the reduced genomes (except in the tree produced using MrBayes where almost all branches were fully supported (posterior probability: 1; Fig. S4).

### Basal biosynthetic capacities

**Fig 4:**
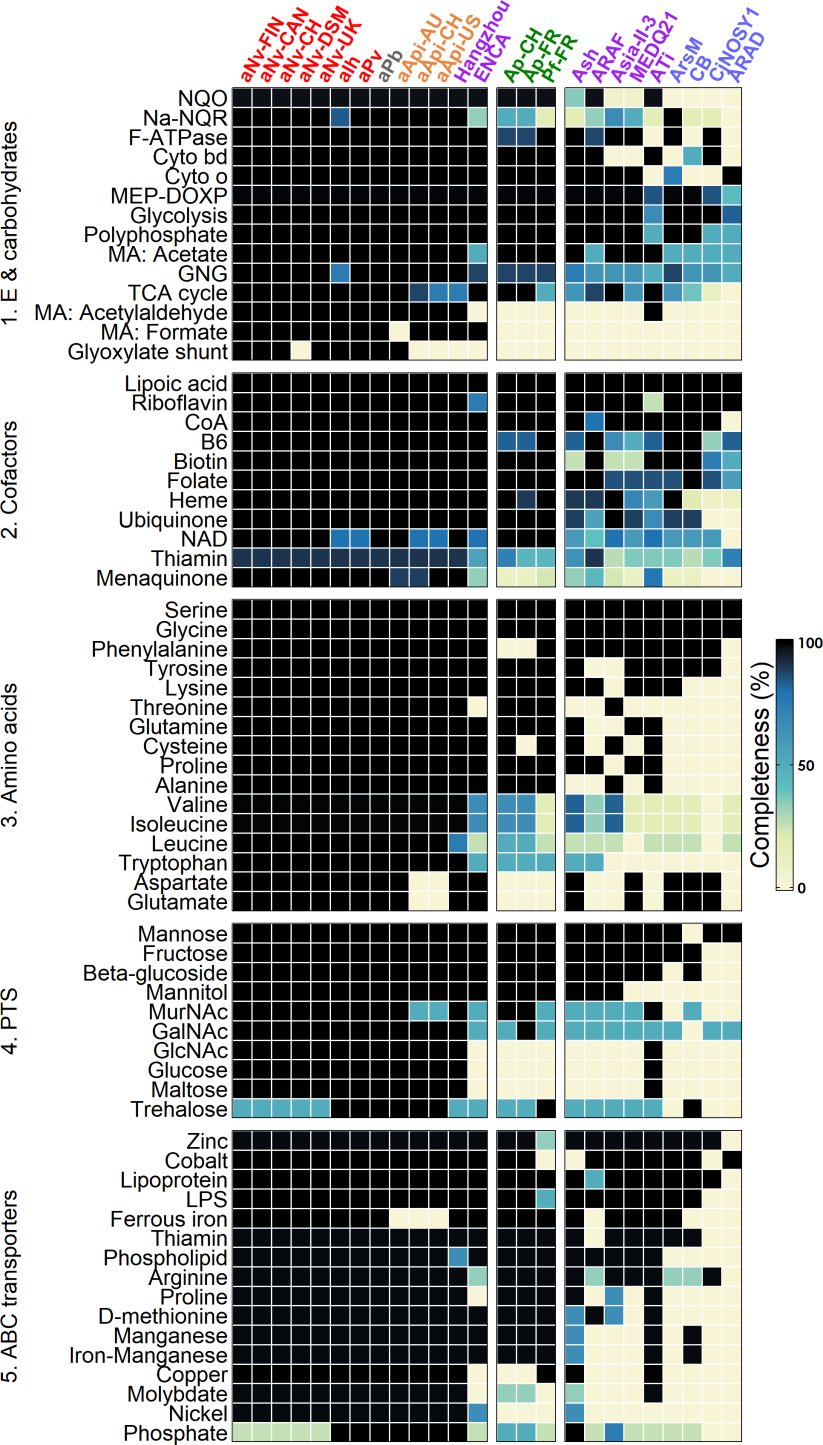
Biosynthetic capacities and transmembrane transport systems among *Arsenophonus* strains. Rows indicate the pathway products or the transported substrates, while columns indicate the strains. Colors indicate the completeness according to the KEGG database. Only pathways complete in at least one strain are shown. Strains are color-coded as in Fig. 1B. B6: vitamin B6; Coa: coenzyme A; Cyto bd: cytochrome bd complex; Cyto O: cytochrome O ubiquinol oxidase; NaNQR: Na^+^-NADH-ubiquinone oxidoreductase; NAD: nicotinamide adenine dinucleotide; NQO: NADH quinone oxidoreductase; MA: mixed acid fermentations; GNG: gluconeogenesis GlcNAc; N-acetylglucosamine. GalNAc: N-acetylgalactosamine. N-acetylmuramic acid: MurNAc; LPS: lipopolysaccharide. MEX-DOXP: non-mevalonate pathway.

The diversity of lifestyles and genome sizes among *Arsenophonus* strains is reflected by substantial variations in basal biosynthetic capacities (Fig. 4, predicted based on Kyoto Encyclopedia of Genes and Genomes [KEGG] pathways). There is a clear erosion gradient from the well-furnished genomes of the culturable strains from clade I to the reduced genomes of P-endosymbionts. Notably, the strain Hangzhou from the *Triatominarum* clade exhibits a basal biosynthetic repertoire almost equivalent to that of clade I strains, consistent with the idea that the genus ancestor was a free-living insect-associated bacterium (37). Ap and Pf have experienced an intermediate level of erosion and have lost many biosynthetic pathways. Nevertheless, these strains have retained more capacities than most members of the *Triatominarum* clade, showing a repertoire highly similar to the strain ENCA. However, considering that both genomes are incomplete, it is possible that their metabolic repertoires were underestimated.

In terms of energy transfer, Ap and Pf have intact NADH quinone oxidoreductase and cytochromes, but the F-type ATPase is incomplete in the Ap strains. For the carbohydrate metabolism, both Ap and Pf can rely on the glycolysis and non-mevalonate pathway, and the Ap strains also have a complete tricarboxylic acid cycle. Interestingly, the phytopathogenic strains have retained the capacity to ferment sugars into acetate, while they have lost other mixed acid pathways. Ap and Pf can produce most cofactors, and this capacity could be involved in nutritional symbiosis with their cixiid hosts feeding on nutrient-limited sap (39, 56). On the other hand, the phytopathogenic strains have lost the ability to produce numerous amino acids, indicating that these compounds must be obtained from the hosts. In particular, glutamate and aspartate might be easily obtained from plants as they represent the most abundant amino acids in the phloem sap (57). Ap and Pf encode several phosphotransferase systems, many ABC transporters, and a siderophore (Fig. 4; Table S4). This capacity for nutrient acquisition could create phloem imbalances and partially explain the plant symptoms associated with SBR and SMC, as suggested for other phloem-infecting bacteria (58–60).

### Genes for virulence and symbiosis

**Fig 5:**
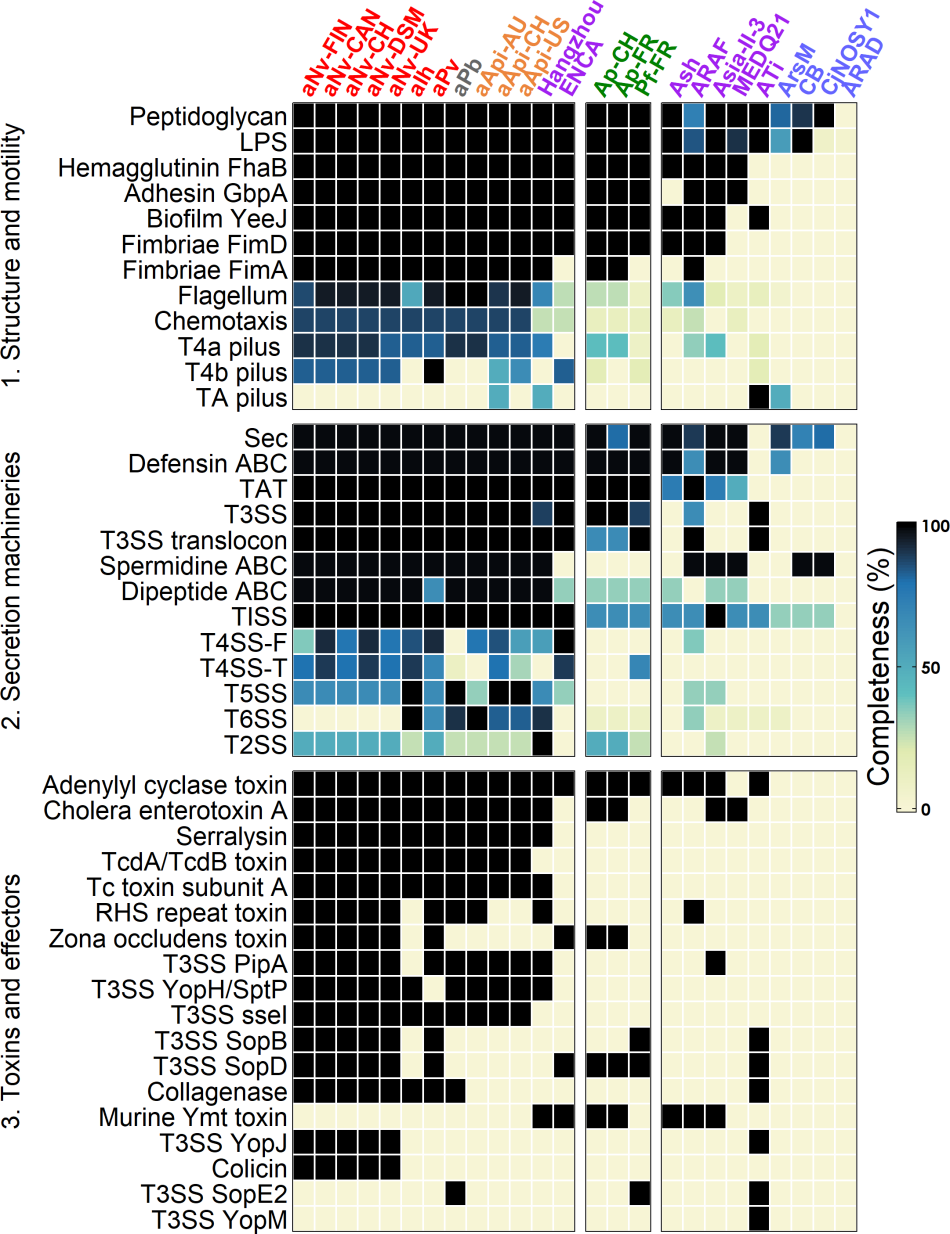
Gene arsenal involved in virulence/symbiosis in the *Arsenophonus* strains. Rows indicate the pathway/protein products, and columns represent the strains. Colors indicate the pathway completeness according to the KEGG database. Only pathways complete or near-complete in at least one strain are shown. Strains are color-coded as in Fig. 1B. TA: tight adherence; TAT: twin arginine translocation; Sec: general secretion; SS: secretion system.

Previous studies have evidenced a complex genetic arsenal dedicated to the virulence and symbiotic lifestyle of *Arsenophonus* strains in their insect hosts (43, 45). Like for the basal capacities, a gradient in this gene arsenal is observed among the strains (Fig. 5). As facultative endosymbionts, Ap and Pf have retained a large portion of this arsenal, which is likely required during the infection of cixiids in order to survive in the gut, cross cellular barriers, evade immunity, and reach the eggs and salivary glands (45).

Ap and Pf possess the genetic capacities to produce lipopolysaccharides and peptidoglycan, consistent with studies reporting typical gram-negative cell walls (61–63). Both strains also harbor genes involved in adhesion but lack the capacity for flagellum, pilus, and chemotaxis, which may explain their fastidious *in planta* multiplication (14, 63). The pathways for general secretion and twin arginine translocation are both conserved in Ap and Pf. These bacteria also have a defensin ABC transporter, and the type 3 secretion system (T3SS) is complete for both Ap strains and almost complete for Pf-FR (lacking only *sctQ*). Conversely, other secretion systems appear absent or incomplete. A few toxins and effectors are predicted in Ap and Pf genomes, and those probably play a role during the insect stage since they are also encoded by non-phytopathogenic strains.

### Genes specific to Ap and Pf

**Fig 6:**
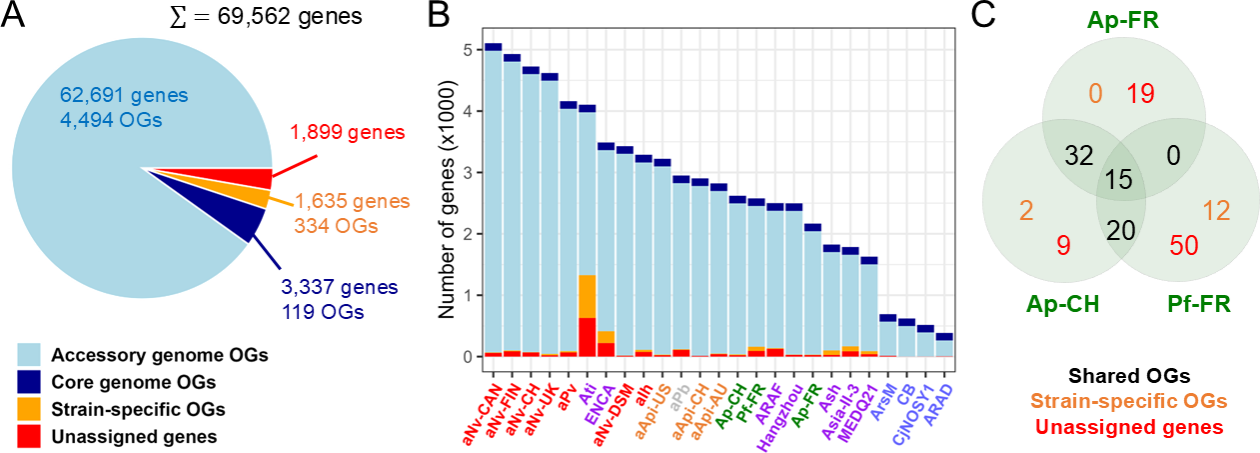
Identification of genes specific to Ap and Pf in the genus. (**A**) Orthology clustering of the *Arsenophonus* pangenome. (**B**) Distribution of genes in each *Arsenophonus* strain. Strains are color-coded as in Fig. 1B. (**C**) Venn diagram of genes only found in Ap and Pf.

The molecular mechanisms underlying the phytopathogenicity of Ap and Pf have not been studied, and it is unclear whether they use similar or distinct strategies. Nevertheless, considering the unique lifestyle of Ap and Pf in the genus, it is imaginable that the genes involved in phloem colonization are lacking in other *Arsenophonus* strains. To identify such “phytopathogen-specific” genes, an orthology clustering analysis was first conducted, resulting in 93% of the *Arsenophonus* pangenome clustered into 4,947 orthogroups (OGs) (Fig. 6A). The core-genome was relatively small, comprising only 3,337 genes in 119 OGs. Importantly, with the exception of ATi and to a lesser extent ENCA, the proportions of strain-specific genes (i.e., unassigned genes and genes in strain-specific OGs) were quite low in all strains (Fig. 6B). Only 159 genes were specific to Ap and/or Pf (Fig. 6C). Of these, some represent regulatory and mobile genetic elements, but the majority encode short hypothetical proteins (HPs) with no known homolog or distant homologs in diverse enterobacteria (Table S5).

**Fig 7:**
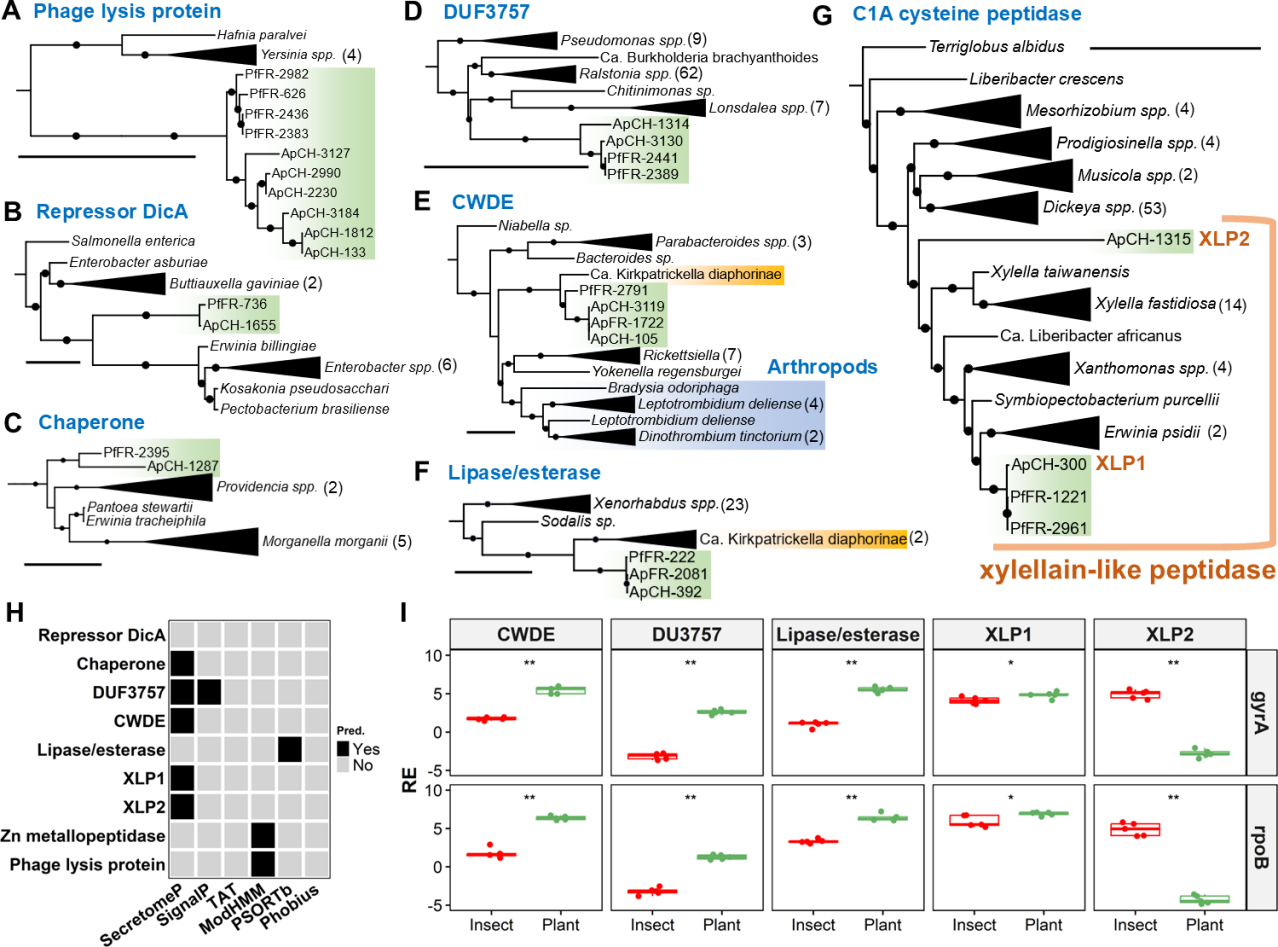
Characterization of a subset of OGs specific to Ap and Pf in the *Arsenophonus* pangenome. (**A–G**) Maximum-likelihood phylogenetic trees. Only the closest homologs are shown in the trees. Black circles on branches indicate >50% bootstrap support. The black scale bars represent one substitution per site. Numbers in brackets indicate the number of genes present in the collapsed clades. *Arsenophonus* genes are highlighted in green. Arthropod genes are highlighted in blue. Genes from *Ca*. K. diaphorinae are highlighted in yellow. The trees were built using the models WAG + I + G4, JTT + I + G4, JTT + G4, VT + I + G4, LG + F + I + G4, VT + I + G4, and WAG + I + G4, respectively. (H) *In silico* predictions for secretion and subcellular localization signals. (I) Box-plots of relative expression (in log2) for selected genes of Ap-CH in insect (red) and plant (green) tissues. Expression levels (*n* = 5) are given relative to the expression of *gyrA* (top) or *rpoB* (bottom). Single and double asterisks indicate *P* < 0.05 and < 0.01, respectively, based on Wilcoxon Rank Sum tests.

Importantly, eight OGs containing 35 genes were exclusively shared by Pf-FR and Ap-CH/FR in the genus (Fig. 6C; Table 2). Proteins from OG3381 are short Zn metallopeptidases with no known homolog. OG2682 corresponds to phage lysis proteins with distant homologs in diverse enterobacteria (Fig. 7A). OG3842 are homologs of the transcriptional repressor *DicA*, with the closest homologs found in *Enterobacter*, the endophyte *Kosakonia pseudosacchari* and the two phytopathogens *Erwinia billingiae* and *Pectobacterium brasiliense* (Fig. 7B). Likewise, OG4231 are putative chaperones with homologs found in both phytopathogenic (*Erwinia tracheiphila* and *Pantoea stewartii*) and non-phytopathogenic enterobacteria (Fig. 7C). Proteins from OG3860 contain the Domain of Unknown Function 3757 (DUF3757) with distant homologs in the phytopathogenic genera *Ralstonia*, *Pseudomonas*, *Lonsdalea*, and *Burkholderia* (Fig. 7D). Similar homologs were also identified among unassigned genes (ApCH_1314 and PfFR_1763, Table S5).

**TABLE 2:** Description of the OGs shared exclusively by Ap and Pf among *Arsenophonus* strains^*a*^

OG	Size (aa)	Domain (*E* value)	Annotation	Best BlastP hit			
				Accession	Size (aa)	ID (%)	Species/strain
2682	150	PF03245 (6^e−27^)	Phage Rz lysis protein	WP_249540969.1	154	41.2	*Escherichia coli* E15
3381	109	COG2321 (2^e−3^)	Zn metallopeptidase	No hit			
3615	533	PF14587 (1.2^e−11^)	O-glycosyl hydrolase	WP_319806414.1	532	86.5	*Ca*. K. diaphorinae
3842	132	PRK09706 (6^e−8^)	Repressor DicA	WP_265910051.1	240	60.0	*E. billingiae* LS-1
3860	142	DUF3757 (3^e−17^)	Unknown	CBJ39888.1	140	53.0	*Ralstonia solanacearum* CMR15
3863	271	COG4870 (2^e−45^)	C1A cysteine peptidase	WP_233592545.1	271	76.7	*E. psidii* LPF 681
3866	504	PF09994 (3^e−18^)	Alpha/beta hydrolase	WP_319806123.1	590	40.4	*Ca*. K. diaphorinae
4231	99	COG2833 (9.2^e−1^)	Molecular chaperone	WP_233480985.1	96	73.7	*E. tracheiphila* MDcuke

^*a*^ Blastp results are shown for the longest gene of each OG.

Curiously, OG3615, OG3866, and two unassigned genes from Pf show homology to genes from the psyllid endosymbiont *Ca*. Kirkpatrickella diaphorinae (64). OG3615 encodes O-glycosyl hydrolases of the CAZyme family GH30. The closest homologs are found in the poorly characterized subfamily GH30-6 (65), with the sole characterized member belonging to *Parabacteroides gordonii* (WP_028726386.1). This enzyme is active on pNP-β-D-cellobioside (66), indicating that OG3615 could represent cell wall-degrading enzymes (CWDEs) targeting plant polysaccharides. Intriguingly, homologs are encoded by arthropod endosymbionts (*Yokenella* and *Rickettsiella*), gut commensals (*Parabacteroides* and *Bacteroides*), and also a fungus gnat (*Bradysia odoriphaga*) and several mites of the genera *Leptotrombidium* and *Dinothrombium* (Fig. 7E). OG3866 represents hydrolases of unknown function distantly related to proteins from diverse non-phytopathogenic enterobacteria (Fig. 7F). An unassigned gene from Pf-FR (PfFR_3139) is also closely related to this OG. These hydrolases putatively represent lipases/esterases as they carry the motif “GxSxG” (67).

Strikingly, OG3863 represents a set of C1A cysteine peptidases closely related to xylellain (AE003869, 65% aa id.), a papain-like peptidase described in *Xylella fastidiosa* (68). Hence, OG3863 and related proteins are hereafter dubbed “xylellain-like peptidases” (XLPs). Ap-CH harbors one version of XLP (ApCH_300, “XLP1” in Fig. 7G) that is highly similar to two copies present in Pf-FR (PfFR_1221 and PfFR_2961). A second, distinct XLP was found among the unassigned genes of Ap-CH (ApCH_1315, “XLP2”), and two additional XLP pseudogenes were found in Pf-FR (PfFR_663 and PfFR_664). Importantly, we detected XLPs in distantly related phytopathogenic *Pseudomonadota*: *Erwinia psidii*, *X. fastidiosa and X. taiwanensis*, *Xanthomonas albilineans* and “pseudalbilineans,” and *Ca*. Liberibacter africanus (Fig. 7G). A homolog is also encoded by the leafhopper endosymbiont *Symbiopectobacterium purcellii*. The XLPs are closely related to enzymes from the phytopathogenic genera *Dickeya* and *Musicola* and from pectinolytic bacteria of the genus *Prodigiosinella* (69). Distant homologs are found in the endophyte *Liberibacter crescens* and nodule-forming *Mesorhizobium* (Fig. 7G).

Given their possible roles during the plant stage of Ap and Pf, the genomic context of the CWDE and XLP genes of Ap and Pf was further analyzed, revealing their presence inside or in close proximity of phage regions (Table S6; Fig. S5 and S6). Interestingly, a CWDE is found on Ap-CH scaffold 9, the putative phage-plasmid element harboring APSE replicative modules. Strikingly, a cluster of nine genes is shared between Ap-CH scaffold 14 and Pf-FR scaffold 18 in proximity of XLP genes. This cluster comprises a phage antirepressor, several HPs, and genes involved in phage integration/excision (excisionase, recombinase, and integrase; Fig. S6). Additional BLAST searches on this cluster indicated similar genes on chromosomes and plasmids in multiple strains of *A. nasoniae* and *A. apicola* (data not shown). Hence, genes from this cluster seem to have propagated across multiple *Arsenophonus* species, and it is tempting to speculate that they mediated the exchange of XLP between Ap and Pf.

### *In silico* predictions and gene expression analyses

Several *in silico* prediction tools were used to assess potential secretion and subcellular localization for the longest representative of each OG exclusively shared by Ap and Pf (Fig. 7H). A non-classical secretion was predicted by SecretomeP for the chaperone, CWDE, XLPs, and DUF3757. SignalP detected a signal peptide for the DUF3757 product, indicating a putative Sec-dependent secretion. Transmembrane regions were evidenced by ModHMM in the Zn metallopeptidase and phage lysis protein, and PSORTb predicted a localization in the outer membrane for the lipase/esterase.

The expression levels of several OGs, in particular the hydrolytic enzymes and DUF3757, were determined by real-time quantitative PCR (RT-qPCR) analyses on RNA samples extracted from Ap-infected stems of periwinkle (*Catharanthus roseus*) and female *P. leporinus* bodies (Fig. 7I). Importantly, expression profiles for all tested genes were similar regardless of the house-keeping gene used as reference (*gyrA* or *rpoB*). Significantly higher expressions *in planta* were detected for the DUF3757, lipase/esterase and CWDE (Wilcoxon rank sum test, all *P* < 0.05), and to a lesser extent XLP1 (Wilcoxon rank sum test, *P* < 0.01). In contrast, XLP2 expression was much higher in the insects than *in planta* (Wilcoxon rank sum test, *P* < 0.05).

## DISCUSSION

For this first genomic analysis of Ap and Pf, three assemblies were obtained from planthopper metagenomes. Despite the use of long-read sequencing, the genomes remain at the scaffold level, partly due to the presence of repetitive viral sequences. Axenic cultures of Ap and Pf will probably be required to obtain longer sequences and achieve gap closure, and this will be valuable to further explore the putative phage-plasmids. Specifically, the APSE modules deserve more scrutiny as they seem widespread in *Arsenophonus* and are known to play important biological roles in other insect endosymbionts (70, 71).

Comparisons of global genomic features and biosynthetic capacities indicate that Ap and Pf are highly similar to several other *Arsenophonus* strains, especially facultative endosymbionts of sap-sucking hemipterans. Our results show that Ap and Pf harbor very low fractions of strain-specific genes, suggesting that the shift from an insect-only to a dual insect-phytopathogenic lifestyle occurred with only a few gene changes. Phylogenomic and ANI analyses further support a recent shift at least for Ap, as evidenced by its very close relationship with other strains from the *Triatominarum* clade. The situation may be more complex for Pf, which represents a distinct species basal to clade I with no other closely related strains known to date. Therefore, it will be interesting to see whether Pf-like strains will be detected in other insect or plant hosts in future research. Importantly, Ap and Pf share a small set of eight OGs unique in the *Arsenophonus* pangenome. Of these OGs, little can be speculated about the putative chaperones, phage lysis proteins, metallopeptidases, DUF3757, and lipases/esterases, although similar proteins have been previously characterized as virulence factors in bacterial phytopathogens (72–74). In contrast, a role in the plant stage of Ap and Pf can be more confidently assumed for the putative CWDEs and XLPs.

The shared CWDEs belong to the large glycoside hydrolase family GH30, which includes enzymes used by phytopathogens to degrade cellulose, hemicellulose, and pectin (75–77). Both Ap and Pf cause physiological alterations in plants including SE obstruction (61, 63), which might be targeted by the CWDEs to facilitate systemic invasion. These enzymes might also have nutritional functions through the degradation of plant polysaccharides into consumable forms. This is supported by the presence of homologs in other arthropod endosymbionts and gut commensals. Surprisingly, homologs of these CWDEs are also found in several mites and a fungus gnat, indicating probable cross-kingdom HGT. Notably, HGT between bacteria and arthropods has been previously documented and includes genes crucial for plant adaptation (78, 79).

The occurrence of XLPs in diverse phytopathogenic *Pseudomonadota* is reported here for the first time and clearly suggests a role of these enzymes in plant tissue colonization. Although a xylellain from *X. fastidiosa* has been purified and its structure resolved, its substrate remains unknown (68). The XLPs represent a sister clade of enzymes found in soft rot agents, endophytes and rhizobia, suggesting that plant proteins represent targets of all these C1A cysteine peptidases. Importantly, all XLP-encoding phytopathogenic bacteria infect the xylem, phloem, or both tissues (80–83). Therefore, these enzymes may specifically act on vasculature proteins. The XLPs are hypothetically secreted, similar to several different cysteine peptidases characterized in other phytopathogens, which are T3SS effectors targeting the plant immunity (84). However, low homology between these effectors and XLPs precludes further comparisons. Intriguingly, an XLP is found in *S. purcellii* SyEd1T, a leafhopper endosymbiont with no known plant stage (85). It should be noted that the closely related strain BEV is plant-transmitted (86), and similar strains have been recently detected in potato (87), indicating that SyEd1T might also have a plant stage. Interestingly, in contrast to Ap XLP1, Ap XLP2 appears to be more expressed in insect than plant tissues. This could be associated with a specific role in the plant adaptation of the cixiid vector through the digestion of phloem proteins, as hypothesized for peptidases encoded by sap-sucking hemipterans and their endosymbionts (88–90). Notably, most if not all the phytopathogenic bacteria encoding XLPs are insect-transmitted, suggesting that XLPs could have multiple roles, both for plant colonization and for the insect vector.

It is likely that some OGs found exclusively in Ap and Pf were transferred from one strain to the another by direct HGT, which is further supported for the XLPs given their shared genomic context. These HGT events might have taken place in a co-infected *C. wagneri* specimen as this planthopper can host both phytopathogens. Prior to these exchanges, some shared OGs were likely acquired from other *Pseudomonadota* (Fig. 7A through G). Importantly, Ap and Pf might have developed the ability to survive in the phloem before acquiring phytopathogenic traits. Indeed, diverse non-phytopathogenic endosymbionts use SEs for horizontal transmission between hemipterans (91–94), and such a plant-mediated transmission would explain the diversity of sap-sucking insects harboring closely related strains of the *Triatominarum* clade. Through this capacity to survive in the phloem, Ap and/or Pf might have been in contact with and acquired genes (e.g., XLP) from other phytopathogens. Noteworthily, several genes seem to have been exchanged with *Ca*. K. diaphorinae, an endosymbiont of the *Liberibacter* vector *Diaphorina citri*. HGT with this bacterium could have occurred in a co-infected hemipteran. HGT *in planta* is also imaginable, as *Ca*. K. diaphorinae is closely related to *Asaia*, a genus of arthropod endosymbionts horizontally transmitted via plants (95, 96). Importantly, to date, the functional roles of most *Arsenophonus* strains from sap-feeding Hemipterans remain unexplored, with only a few documented exceptions (31–33, 39). This suggests the possibility that other strains may have the potential to induce unidentified plant diseases. Therefore, further investigation into the biology and ecology of these poorly characterized strains from the *Triatominarum* clade is imperative.

In conclusion, our results strengthen the idea of an “insect first” scenario during the evolution of Ap and Pf toward becoming vector-borne phytopathogens. A limited number of HGT events probably acted as key mechanisms in this lifestyle transition, which aligns with recent studies associating HGT with the shift from a non-vascular to a vascular phytopathogen (97, 98). In particular, the acquisition and exchange of CWDEs and XLPs might underlie the double emergence of phytopathogenicity in *Arsenophonus*. Biochemical characterization of these enzymes and their substrates will be required to further support this hypothesis.

## MATERIALS AND METHODS

### DNA extraction for insect metagenomes

For Pf-FR, adult *C. wagneri* specimens were collected in a strawberry field in Dordogne (France) in June 2019. For Ap-FR, adult *P. leporinus* specimens were collected during the first SBR outbreak in Burgundy (France) in the early 2000s. For Ap-CH, adult *P. leporinus* specimens were collected in a sugar beet field in Gilly (Switzerland) in 2020. Insects from France were surface sterilized by serial washes in 20% bleach, sterile water, 70% ethanol, and sterile water. Individual insects were then ground in 400 µL 2% cetyltrimethylammonium bromide (CTAB) buffer (2% CTAB, 2% polyvinylpyrrolidone [PVP] K40, 1.4 M NaCl, 20 mM EDTA, 100 mM Tris-HCl, and 0.02% β-mercaptoethanol) and incubated at 65°C for 1.5 hours. The DNA was then extracted as previously described (63). No surface sterilization was performed for insects collected in Switzerland, and they were stored in 70% ethanol at −20°C until further use. For these specimens, DNA was extracted using a 3% CTAB protocol (14). All DNA samples were treated with 20 µg RNase A at 37°C for 30 min, followed by chloroform/isoamyl alcohol extraction and isopropanol precipitation. DNA pellets were resuspended in 40 µL of 1× TE buffer for samples from France, whereas 100 µL of DNase-free water was used for Swiss samples.

### Quantification of bacterial titre

Bacterial titers were estimated in DNA samples by qPCR. For Pf-FR and Ap-FR, samples were normalized to 50 ng/µL to compare Ct values between samples. All samples were tested in duplicates with the previously published primers and FAM-labeled TaqMan probes targeting the *spoT* gene of Pf (4). For Ap-FR, primers and probe (SBR-F/R/FAM) are listed in Table S1. Reactions were performed as previously described (63). For samples from Switzerland, a similar qPCR assay was performed using a different protocol (14). Samples with the highest bacterial titers were chosen for high-throughput sequencing.

### Genome sequencing and assembly

For Ap-CH and Pf-FR, long-read sequencing libraries were prepared using the Ligation Sequencing kits SQK-LSK 110 and 109 (Oxford Nanopore Technologies, UK), respectively. Each library was sequenced on an R9.4 flow cell on the MinION sequencer, producing 16 and 6 Gb of data, respectively. Base calling was done using Guppy v5.0.11 (in high-accuracy mode) and only ≥500 bp reads passing the Q7 quality filter were retained. In addition, 2 × 150 bp paired-end reads were obtained from an Illumina Novaseq platform (Macrogen), producing 50 and 476 million reads for Ap-CH and Pf-FR, respectively. The reads were quality-trimmed using Trimmomatic v0.38 (99), retaining only reads ≥Q30.

Reads belonging to *Arsenophonus* were extracted from the data sets via mapping (using Minimap2 v2.15 [100]) for the long reads and bwa mem v0.7.17 for the short reads, respectively) against a database of all published *Arsenophonus* genomes (as of September 2023; Table 1). Several hybrid assemblers were then tested: Masurca v4.0.7 (101), Spades v3.15.1 (102), and Unicycler v0.4.9 (103). For Pf-FR, the most contiguous assembly was obtained using Masurca and was further polished with short reads using Polca (part of the Masurca package) and scaffolded using two iterations of SSPACE v2.1.1 (104). For Ap-CH, the most contiguous assembly was obtained using Unicycler, and this assembly was also scaffolded using two iterations of SSPACE.

The genome of Ap-FR was assembled only from short reads since the samples had been stored for more than 15 years prior to DNA extraction, resulting in highly fragmented DNA precluding long-read sequencing. Illumina sequencing was performed as mentioned above, producing 486 million 150 bp paired-end reads. Following quality trimming, the reads were assembled using Megahit v1.2.9 (105) and contigs belonging to Ap were identified using BlobTools v1.1.1 (https://github.com/DRL/blobtools) and mapping against a database of all published *Arsenophonus* genomes using bwa mem. The contigs were scaffolded with Redundans (106) using the Ap-CH assembly as reference, followed by one iteration of SSPACE and Gapfiller v2.1.2 (107). Coverages were determined with Mosdepth v0.3.4 (108). CheckM v1.1.6 (109) was used to assess genome completeness. Synteny blocks were identified with Sibelia v3.0.7 (110).

### Functional annotation

The three assemblies were annotated using the National Center for Biotechnology Information (NCBI) PGAP v2023-05-17.build6771. Clusters of Orthologous Groups (COG) categories and KEGG ontology terms were determined using eggNOG-mapper v2 (111). Phage regions were detected with Phaster (112). Schematic representations of genetic modules were obtained with Clinker (113). Circular representations of the scaffolds were produced with the R package Circlize v0.4.10 (114). Heatmaps were constructed using the ComplexHeatmap R package (115). Biosynthetic capacities were evaluated using KEGG Decoder v1.3 (116), TXSscan v1.1.0 (117), and antiSMASH v7.beta (118). Toxins and effectors were detected by Blastp searches in a local database of *Arsenophonus* proteomes.

### Phylogenomics and taxonomy

Orthofinder v2.5.4 (119) was used to identify single-copy orthologs shared between Pf-FR, Ap-CH, Ap-FR, and 22 *Arsenophonus* genomes published as of September 2023 (Table 1). *Proteus mirabilis* HI4320 (GCF_000069965.1) and *Providencia stuartii* MRSN 2154 (GCF_000259175.1) were used as outgroups. Amino acid sequences of each gene were aligned using Muscle v3.1.31 (120), followed by concatenation with geneStitcher.py (https://github.com/ballesterus/Utensils/blob/master/geneStitcher.py). The resulting multi-gene matrix was analyzed using three different phylogenetic methods: (i) IQ-TREE v1.6.12 (121) was used to predict the best substitution model for each gene partition (122, 123) and to produce a maximum likelihood phylogenetic tree with 1,000 bootstrap iterations. The tree was edited in iTOL (124). (ii) A Bayesian phylogenetic analysis was performed using MrBayes v3.2.7 (125). The consensus tree was established from two independent runs, each with four chains (three hot and one cold chain) run for 3,000,000 generations. Trees were sampled every 1,000 generations with 25% burn-in. The best substitution model was determined by MrBayes (aamodelpr = mixed), with a gamma-distributed rate variation across sites (four discrete gamma categories) and a proportion of invariable sites (invgamma). (iii) Considering that some *Arsenophonus* strains are known to create extremely long branches (126), we performed a second Bayesian analysis using PhyloBayes-MPI v1.9 (127) with the CAT-GTR model to minimize the impact of long-branch attractions. Two separate chains were run for >10,000 generations, and convergence was assessed using the bpcomp and tracecomp functions (maxdiff < 0.3 and effect size > 50). The same phylogenetic analyses were performed including also “*Candidatus* Riesia” as well as several other insect endosymbionts from the *Enterobacteriaceae* family (*Buchnera aphidicola*, *H. defensa*, and *Wigglesworthia glossinidia*), in line with *Riesia*’s current taxonomic assignment and the phylogenetic analyses by Boyd et al. (128). These analyses suggested that the frequently observed clustering of *Riesia* with some *Arsenophonus* P-endosymbionts might be an artifact caused by long-branch attractions (Fig. S7 to S9). Therefore, *Riesia* was not included in the comparative analyses presented herein.

ANI values were obtained using the enveomics toolbox collection (129).

### Pangenome analysis

An orthology clustering analysis was conducted using Orthofinder v2.5.4 (119) on all *Arsenophonus* genomes used for the phylogenomic analyses. Shared OGs were identified using the R package UpSetR v1.4.0 (130). To further analyze the genes present only in Ap and/or Pf, Blastp searches were conducted on the longest sequence for each OG. These searches excluded additional undetected pseudogenes and orthologs shared with recently published *Arsenophonus* genomes reconstructed from Sequence Read Archive (SRA) data sets and endosymbionts of louse flies (43, 126). For the OGs shared by Ap and Pf, functional domains were identified with MotifFinder (https://www.genome.jp/tools/motif/). *In silico* predictions were conducted using SignalP v6.0 (131), SecretomeP v2.0 (132), and PSORTb v3.0.3 (133). Phylogenetic analyses were performed as previously described (134).

### Gene expression analyses

RT-qPCR analyses were conducted for several Ap-CH genes in *P. leporinus* females and periwinkle stems. Insects were collected at the adult stage, after a rearing period of *c*. 5 months in controlled conditions (135). Periwinkle seedlings were inoculated with field-collected insects as previously described (14) and then maintained in insect-proof cages in greenhouse conditions for 3 months. Whole insect bodies and plant stem samples were freeze-dried in liquid nitrogen and stored at –80°C until further use. Total RNA was extracted using a 3% CTAB protocol (14), treated with DNase, and quantified using a Qubit fluorometer. Complementary DNA (cDNA) was synthetized for 1 µg of each RNA sample using random hexamers (Invitrogen) in combination with the Superscript reverse transcriptase (Promega). qPCR assays were conducted as previously described (14), using primers and probes listed in Table S1. Expressions were obtained for five biological replicates and were normalized using the Ap-CH *gyrase* or *rpoB* genes. Mean relative expressions were compared by Wilcoxon Rank Sum tests in R. qPCR efficiencies were assessed based on five to six 10-fold serial dilutions of an infected sample in healthy RNA extracts. Specificity was checked by gel electrophoresis.

## Data Availability

The genomes produced in this work are accessible in the NCBI database under GenBank accessions GCA_047291315.1 (*Ca*. Phlomobacter fragariae), GCA_047291415.1 (*Ca*. Arsenophonus pathogenicus Ap-CH) and GCA_047291325.1 (*Ca*. Arsenophonus pathogenicus Ap-FR).

## SUPPLEMENTAL MATERIAL

Supplemental figures and tables msystems.01496-24-s0001.pdf Tables S1 to S6 and Fig. S1 to S9.

ASM does not own the copyrights to Supplemental Material that may be linked to, or accessed through, an article. The authors have granted ASM a non-exclusive, world-wide license to publish the Supplemental Material files. Please contact the corresponding author directly for reuse.
