# Supplementary material for: From insect endosymbiont to phloem colonizer: comparative genomics unveils the lifestyle transition of phytopathogenic Arsenophonus strains
Source: mSystems. 2025 Apr 9;10(5):e01496-24. doi: 10.1128/msystems.01496-24 (PMC12090721; doi:10.1128/msystems.01496-24)
Supplement: Supplemental figures and tables — Tables S1 to S6 and Fig. S1 to S9. [file msystems.01496-24-s0001.pdf]

Supplementary tables

Table S1. Primers and probes used in this study.

Use	Target	Name	Sequence
qPCR	SpoT	SBR-F	TATCTACCTGCAAAGCTGTG
		SBR-R	CCCAAGATGATACAAGTAGG
		SBR-FAM	FAM-ATCT*TAG*CCG*CTT*ATCTTAACA*AC-BHQ
RT-qPCR	Gyrase	ApCHgyra2F	GGCGATAAGGTCGTTTCGCTG
		ApCHgyra2R	GTAGCACGAGATTTTGTCGGGT
		Probe-Gyr	FAM-CGTTACAGAAAATGGTTATGGTAAACGAAC-BHQ
	RpoB	ArsRpoB2F	TGGCCGTGGAAGATTGACTGA
		ArsRpoB2R	GCGTACGACTGGCAAAGCAAC
		Probe-rpoB	FAM-AGAAACATTACGTGAAGTTCGCATGGCGT-BHQ
	DUF3757	ApCH3079F	GAAGGTGAAAACCCCATCGC
		ApCH3079R	CATTTGGCACATTCCCGACA
		Probe-3079	FAM-GATTTGAGCGTGGTATCTTTCAGGGAAAGTT-BHQ
	Lipase/ esterase	ApCH1208F	GTTGCGGCCGTTTTAGCTTT
		ApCH1208R	GCGTATTTCATGTCCAGCGG
		Probe-1208	FAM-ACAGGTAGTATCGATATCACCCTTAACAAGGA-BHQ
	CWDE	ApCH3069F	TTGGAGTTCGCCGCTAGTTT
		ApCH3069R	CAACACCAAGCCAGAACGTG
		Probe-3069	FAM-TTGCATAATTTTCTAACAAAAGGGAATATAAGCGCT-BHQ
	XLP1	ApCHXLP1qPCRF	ATGGCCTTATGACGACACAC
		ApCHXLP1qPCRR	TTGCCGAGGAATAACATCCC
		Probe-XLP1	FAM-GCCTCATTCAAGAGCTGGACAGAGT-BHQ
	XLP2	ApCHXLP2qPCRF	TGCCACGCATATTCCCTTAC
		ApCHXLP2qPCRR	AACAGCTGTGTACGGTCATC
		Probe- XLP2	FAM-GGACATGCCGTCCTGTGTGTAGGA-BHQ

* LNA-modified base.

Table S2. Regions of Ap-CH and Pf-FR syntenic to *Arsenophonus* extrachromosomal elements.

Strain	Scaffold:positions	Extrachromosomal elements (accession, positions)
Ap-CH	1:785860-760093	paIh1 (NZ_CP123491.1:18-26042)
	2: 369810-363307	paNv_CAN2 (NZ_CP123525.1 :90465-97042)<br>paNv_CH2 (NZ_CP123546.1 :56695-63272)<br>paNv_UK2 (NZ_CP123563.1 :137329-143906)<br>pArsFIN5NZ (CP038617.1 :79084-85661)
	3: 12996-6803	pArsBee3 (NZ_CP084225.1:32128-25222)<br>paApi-AU2 (NZ_CP123758.1: 25975-32141)
	11:31920-37789	paApi_AU1 (NZ_CP123757.1:75344-82140)<br>paNv_CAN11 (NZ_CP123534.1:29795-36384)<br>paNv_CAN10 (NZ_CP123533.1:21989-28578)<br>paNv_CAN4 (NZ_CP123527.1:36716-29845)<br>paNv_CH8 (NZ_CP123552.1:17598-11009)<br>paNv_CH7 (NZ_CP123551.1:19347-26214)<br>paPv8 (NZ_CP123512.1:16955-23902)<br>paPv7 (NZ_CP123511.1:15995-22942)<br>paPv6 (NZ_CP123510.1:21760-28701)<br>paPv2 (NZ_CP123506.1:7223-13836)<br>paNv_UK9 (NZ_CP123570.1:17571-24418)<br>paNv_UK7 (NZ_CP123568.1:14833-7964)<br>paNv_UK5 (NZ_CP123566.1:26957-20098)<br>pArsFIN5 (NZ_CP038617.1:52413-58128)
Pf-FR	3:93637-148395	paIh1 (NZ_CP123491.1:60968-10)

Table S3. Plasmid genes identified in Ap-CH and Pf-FR.

Strains	Scaffold:positions	Locus	Annotation
Ap-CH	2:358558-358755	APCH_1762	Phage/plasmid replication protein
	2:358891-359262	APCH_1763	Phage/plasmid replication protein, II/X family
	2:363286-364569	APCH_1769	Phage/plasmid replication protein, II/X family
	3:241561-241758	APCH_2080	Phage/plasmid replication protein
	3:241721-241903	APCH_2081	Phage/plasmid replication protein, II/X family
	3:241998-242513	APCH_2083	Phage/plasmid replication protein, II/X family
	9:15299-15517	APCH_3136	Plasmid partition protein ParG
	9:22246-23097	APCH_3149	Plasmid replication initiator TrfA
	11:13478-14434	APCH_0095	Plasmid segregation protein ParM
	11:14442-14801	APCH_0096	Plasmid partitioning/stability family protein
	26:1-230	APCH_1328	Phage/plasmid replication protein, II/X family
Pf-FR	3:121293-121544	PfFR_2423	Plasmid stabilization protein
	4:138477-139433	PfFR_2447	Plasmid segregation protein ParM
	4:139440-139802	PfFR_2448	Plasmid partitioning/stability family protein
	6:79551-80555	PfFR_2765	Plasmid segregation protein ParM
	6:80556-80882	PfFR_2766	Plasmid partitioning/stability family protein
	9:84826-84996	PfFR_3196	Phage/plasmid replication protein, II/X family
	9:91154-91300	PfFR_3204	Phage/plasmid replication protein, II/X family

Table S4. Biosynthetic gene clusters identified by antiSMASH in Ap-CH and Pf-FR.

**Strains**	**Scaffold:positions**	**Type**	**Similarity to known cluster (%)**	**Best homology in *Arsenophonus****
Ap-CH	1:543,657-584,955	NRPS-like	-	Hangzhou (53)
	2:26,406-47,068	Homoserine lactone	-	Hangzhou (100)
	2:208,647-238,497	NI-siderophore	Putrebactin/avaroferrin (30)	Ash (53)
	4:105,818-130,861	Beta-lactone	lomaiviticin A (3)	An-Nv FIN (90)
	12:19,802-31,547	RiPP-like	-	Hangzhou (52)
Pf-FR	1:150,376-192,632	NI-siderophores	Putrebactin/avaroferrin (100)	An-Nv FIN (60)
	5:28,112-39,869	RiPP-like	-	An-Nv FIN (52)
	12:49,996-74,412	Beta-lactone	-	An-Nv FIN (76)
	17:45,374-57,069	Homoserine lactone	-	An-Nv FIN (36)

*Numbers in brackets indicate the percentage of genes showing similarity.

Table S5. Description of the OGs present only in Ap-FR/CH or Pf among *Arsenophonus* strains. Results are shown for the longest gene of each OG.

OG	Locus	Size (aa)	Best BlastP hit				
			Accession	Size (aa)	ID (%)	Annotation	Species
3771	PfFR_623, PfFR_2599, PfFR_2979	172	WP_275372162.1	171	73	HP	Xenorhabdus bovienii
4060	PfFR_656, PfFR_1314, PfFR_2351	56	ELA8071006.1	117	50	HP	Proteus mirabilis
4149	APCH_0432, APFR_0401	53	NA	NA	NA	HP	NA
4155	APCH_0750, APFR_0890	45	MCV5747073.1	56	93	Transcriptional regulator	Escherichia coli
4156	APCH_0751, APFR_0891	42	WP_115825434.1	265	86	Transcriptional regulator	Xenorhabdus cabanillasii
4157	APCH_0752, APFR_0892	55	WP_240229850.1	245	89	Transcriptional regulator	Klebsiella pneumoniae
4158	APCH_0753, APFR_0893	113	WP_228205247.1	265	87	Transcriptional regulator	Serratia ureilytica
4159	APCH_0757, APFR_0897	83	WP_198298551.1	192	82	Endonuclease subunit S	Dickeya dianthicola
4175	APCH_1024, APFR_2567	42	NA	NA	NA	HP	NA
4176	APCH_1045, APFR_2588	82	NA	NA	NA	HP	NA
4195	APCH_1894, APFR_2150	20	NA	NA	NA	HP	NA
4196	APCH_1945, APFR_2202	130	WP_181489803.1	158	62	HP	Providencia sp.
4197	APCH_1946, APFR_2203	78	WP_181489802.1	158	85	HP	Providencia sp.
4209	APCH_2504, APFR_2310	157	WP_237654170.1	194	92	HP	Cronobacter malonaticus
4210	APCH_2505, APFR_2311	113	ELY2553240.1	252	88	HP	Cronobacter sakazakii
4214	APCH_3111, APFR_1912	51	WP_241916208.1	388	46	Chromosome segregation ATPase	Yersinia intermedia
4216	APCH_3145, APFR_1713	161	HCK6986336.1	158	60	DM13 domain-containing protein	Klebsiella pneumoniae
4222	APCH_0114, APFR_1346	45	NA	NA	NA	HP	NA
4653	PfFR_421, PfFR_2911	67	APC14106.1	136	58	Transposase	Providencia rettgeri
4654	PfFR_484, PfFR_1978	116	NA	NA	NA	HP	NA
4655	PfFR_489, PfFR_2206	41	NA	NA	NA	HP	NA
4202	APCH_2303, APCH_2356	43	NA	NA	NA	HP	NA
NA	APCH_0263	208	WP_140585783.1	217	52	AAA family ATPase	Haemophilus haemolyticus
NA	APCH_0264	374	WP_181154750.1	616	45	AAA family ATPase	Haemophilus haemolyticus
NA	APCH_0403	422	WP_091346969.1	422	52	HP	Gilliamella bombicola
NA	APCH_1016	66	NA	NA	NA	HP	NA
NA	APCH_1279	180	WP_125179960.1	656	49	N-6 DNA methylase	Thiohalobacter thiocyanaticus
NA	APCH_1314	122	CAH0447301.1	126	54	DUF3757	Ralstonia syzygii subsp. syzygii
NA	APCH_1315	263	WP_038272339.1	271	49	C1 family peptidase	Xylella taiwanensis
NA	APCH_2292	110	EKW8485287.1	104	59	HP	Morganella morganii
NA	APCH_2293	242	HBA4204091.1	242	73	HP	Escherichia coli
NA	APFR_0189	128	NA	NA	NA	HP	NA
NA	APFR_0590	93	NA	NA	NA	HP	NA
NA	APFR_1317	45	NA	NA	NA	HP	NA
NA	APFR_1347	55	NA	NA	NA	HP	NA
NA	APFR_1356	59	NA	NA	NA	HP	NA
NA	APFR_1558	48	EWE16279.1	67	60	HP	Klebsiella pneumoniae
NA	APFR_1620	65	NA	NA	NA	HP	NA
NA	APFR_1653	49	NA	NA	NA	HP	NA
NA	APFR_1667	43	NA	NA	NA	HP	NA
NA	APFR_1892	56	WP_181681090.1	109	57	HP	Citrobacter sp. RHB25-C09

NA	APFR_1919	41	NA	NA	NA	HP	NA
NA	APFR_1936	62	NA	NA	NA	HP	NA
NA	APFR_1942	43	NA	NA	NA	HP	NA
NA	APFR_2084	73	NA	NA	NA	HP	NA
NA	APFR_2085	52	NA	NA	NA	HP	NA
NA	APFR_2149	49	NA	NA	NA	HP	NA
NA	APFR_2246	48	NA	NA	NA	HP	NA
NA	APFR_2253	68	NA	NA	NA	HP	NA
NA	APFR_2553	110	NA	NA	NA	HP	NA
NA	PfFR_033	59	NA	NA	NA	HP	NA
NA	PfFR_109	46	NA	NA	NA	HP	NA
NA	PfFR_150	56	NA	NA	NA	HP	NA
NA	PfFR_155	73	NA	NA	NA	HP	NA
NA	PfFR_156	45	WP_025203618.1	103	48	HP	Enterobacter ludwigii
NA	PfFR_169	59	NA	NA	NA	HP	NA
NA	PfFR_424	75	NA	NA	NA	HP	NA
NA	PfFR_429	41	NA	NA	NA	HP	NA
NA	PfFR_452	92	NA	NA	NA	HP	NA
NA	PfFR_476	65	NA	NA	NA	HP	NA
NA	PfFR_663	177	MCX8958438.1	280	72	C1 family peptidase	Ewinia psidii
NA	PfFR_664	62	WP_252120354.1	271	86	C1 family peptidase	Symbiopectobacterium purcellii
NA	PfFR_808	15	NA	NA	NA	HP	NA
NA	PfFR_1165	59	NA	NA	NA	HP	NA
NA	PfFR_1236	75	WP_331398216.1	325	62	IS91 family transposase	Enterobacter quasihormaechei
NA	PfFR_1237	57	WP_309929426.1	866	69	Glycosyl hydrolase family 18 protein	Caballeronia sp.
NA	PfFR_1252	184	NA	NA	NA	HP	NA
NA	PfFR_1262	50	WP_275902775.1	52	60	HP	Morganella morganii
NA	PfFR_1392	73	MCV5626576.1	70	47	Glycosyltransferase	Escherichia coli
NA	PfFR_1459	46	WP_099119852.1	119	74	HP	Xenorhabdus sp.
NA	PfFR_1727	21	NA	NA	NA	HP	NA
NA	PfFR_1735	41	NRB41646.1	875	73	Histidinol-phosphatase	Pseudomonadales bacterium
NA	PfFR_1762	37	WP_264370807.1	93	97	type I restriction enzyme M protein	Paraglaciecola polaris
NA	PfFR_1763	42	AMP40542.1	126	51	DUF3757	Ralstonia solanacearum
NA	PfFR_1835	61	CAK8743259.1	72	49	HP	Sodalis praecaptivus
NA	PfFR_1841	56	NA	NA	NA	HP	NA
NA	PfFR_1877	94	HIO24170.1	137	46	Pentapeptide repeat-containing protein	Nitrospinaceae bacterium
NA	PfFR_1887	77	NA	NA	NA	HP	NA
NA	PfFR_1944	198	WP_041175950.1	626	43	E3 ubiquitin--protein ligase	Yersinia pseudotuberculosis
NA	PfFR_1981	85	NA	NA	NA	HP	NA
NA	PfFR_1995	45	NA	NA	NA	HP	NA
NA	PfFR_2003	53	NA	NA	NA	HP	NA
NA	PfFR_2174	54	NA	NA	NA	HP	NA
NA	PfFR_2226	61	NA	NA	NA	HP	NA
NA	PfFR_2228	77	WP_119797362.1	364	41	HP	Ca. Fukatsuia symbiotica
NA	PfFR_2252	42	ELN2576909.1	80	57	AlpA family phage regulatory protein	Enterobacter kobei
NA	PfFR_2262	53	NA	NA	NA	HP	NA

NA	PfFR_2373	68	NA	NA	NA	HP	NA
NA	PfFR_2392	106	WP_272515617.1	101	57	Transcriptional regulator	Unclassified Providencia
NA	PfFR_2508	104	NA	NA	NA	HP	NA
NA	PfFR_2662	53	NA	NA	NA	HP	NA
NA	PfFR_2707	48	BAN97797.1	108	52	HP	Plautia stali symbiont
NA	PfFR_2716	249	WP_319807707.1	149	73	Glycoside hydrolase family 18	Ca. Kirkpatrickella diaphorinae
NA	PfFR_2789	95	RAP72549.1	142	41	HP	Ca. Erwinia dacicola
NA	PfFR_2795	49	NA	NA	NA	HP	NA
NA	PfFR_2813	147	WP_014542758.1	437	69	M20 family metallopeptidase.	Erwinia sp. Ejp617
NA	PfFR_2814	66	WP_038270375.1	436	82	M20 family metallopeptidase	Xylella taiwanensis
NA	PfFR_3139	172	UYH52251.1	208	43	DUF2235	Ca. Kirkpatrickella diaphorinae
NA	PfFR_3140	52	WP_038174582.1	690	51	DUF2235	Vibrio pacinii
NA	PfFR_3188	47	NA	NA	NA	HP	NA

Table S6. Locations of XLP and CWDE genes of Ap-CH/FR and Pf-FR relatively to phage regions.

Locus	Scaffold	Positions	Note
XLPs			
PfFR_1221	23	12330-13145	Next to incomplete phage region 20
PfFR_2961	7	104791-105606	Inside questionable region 11
PfFR_664	18	42497-42685	Inside intact phage region 17
ApCH_300	14	32161-32976	Next to questionable phage region 14
ApCH_1315	24	9086-9877	Next to incomplete phage region 18
CWDEs			
PfFR-2791	6	96120-97721	Not close to any phage region
ApCH-105	11	20445-22034	Inside questionable region 13
ApCH-3119	9	5947-7536	Inside incomplete phage region 10
ApFR-1722	38	1540-3129	-

Supplementary figures

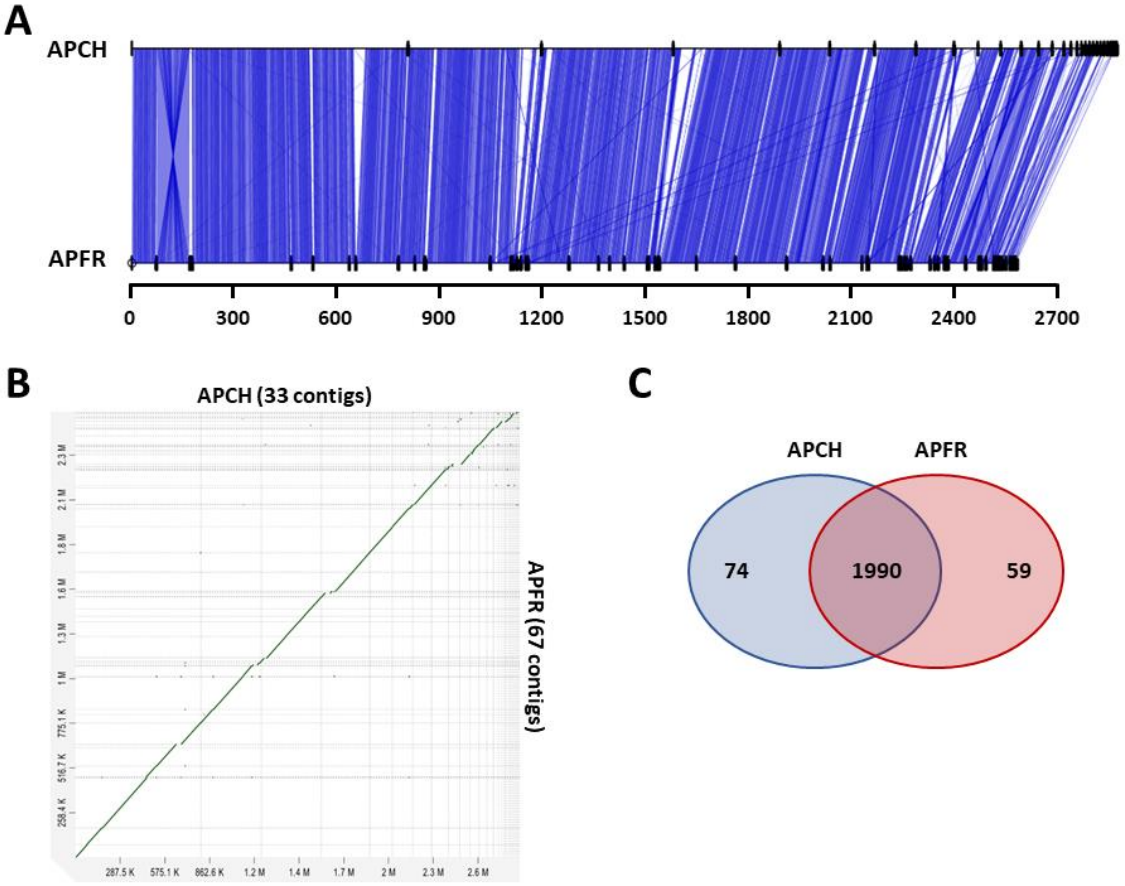

Figure S1. Comparison of Ap-CH and Ap-FR assemblies. **(A)** Synteny between the two Ap genomes. **(B)** Alignment of the scaffolds of Ap-CH and Ap-FR. **(C)** Venn diagram of shared protein-coding genes between Ap-CH and Ap-FR.

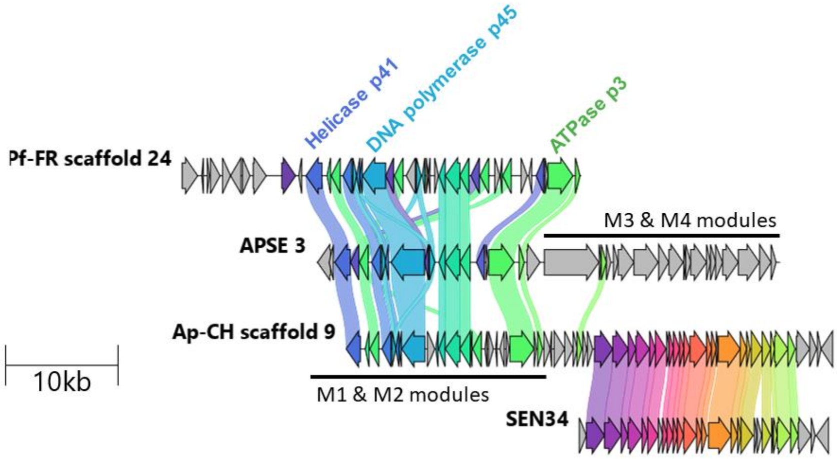

Figure S2. Gene clusters from Ap-CH and Pf-FR syntenic to the APSE modules 1 and 2. The sequence of APSE 3 (LR794150.1) was used as an APSE representative. The rest of Ap-CH scaffold 9 is syntenic to structural genes from Salmonella phage SEN34 (NC_028699.1).

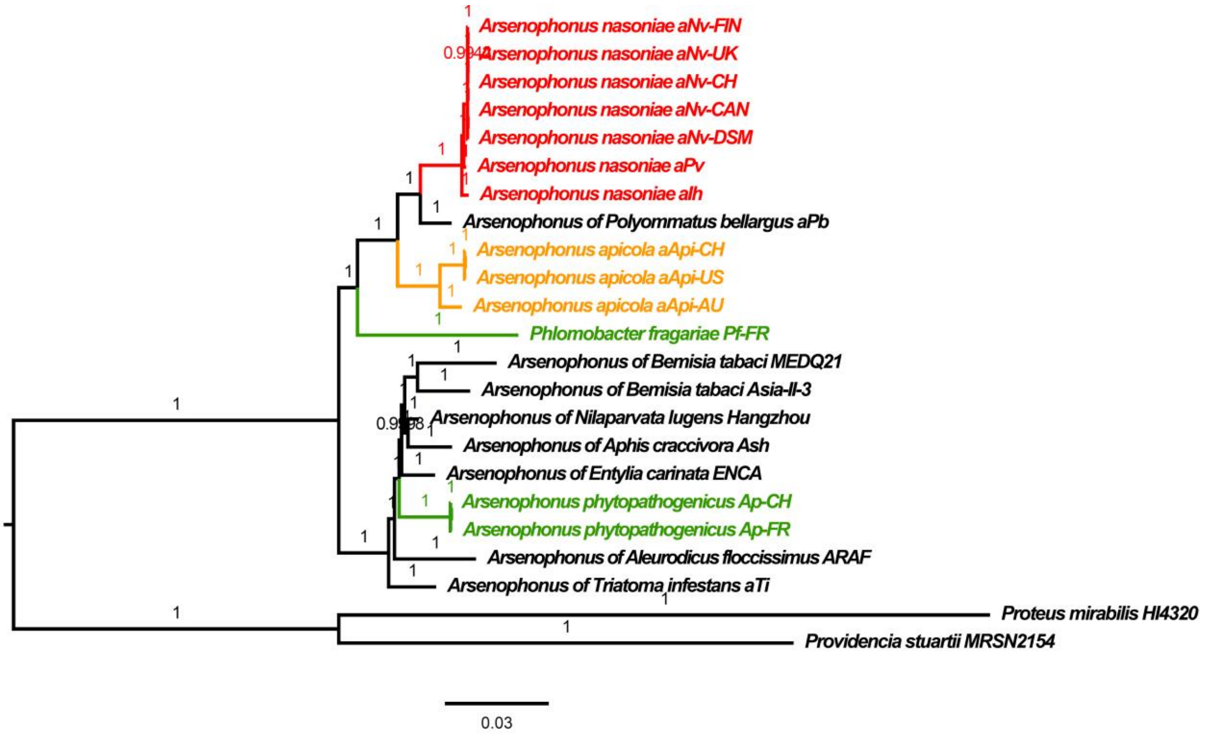

Figure S3. Bayesian phylogenomic analysis of Ap, Pf and facultative *Arsenophonus* strains produced using MrBayes on the same multi-gene alignment as the maximum-likelihood tree presented in Fig. 3. The tree is based on 280 shared single copy protein-coding genes. All branches have posterior probabilities >0.9. Genes from *Providencia stuartii* and *Proteus mirabilis* were used to root the tree. The tree scale represents 0.03 substitutions per site.

(A) Maximum Likelihood

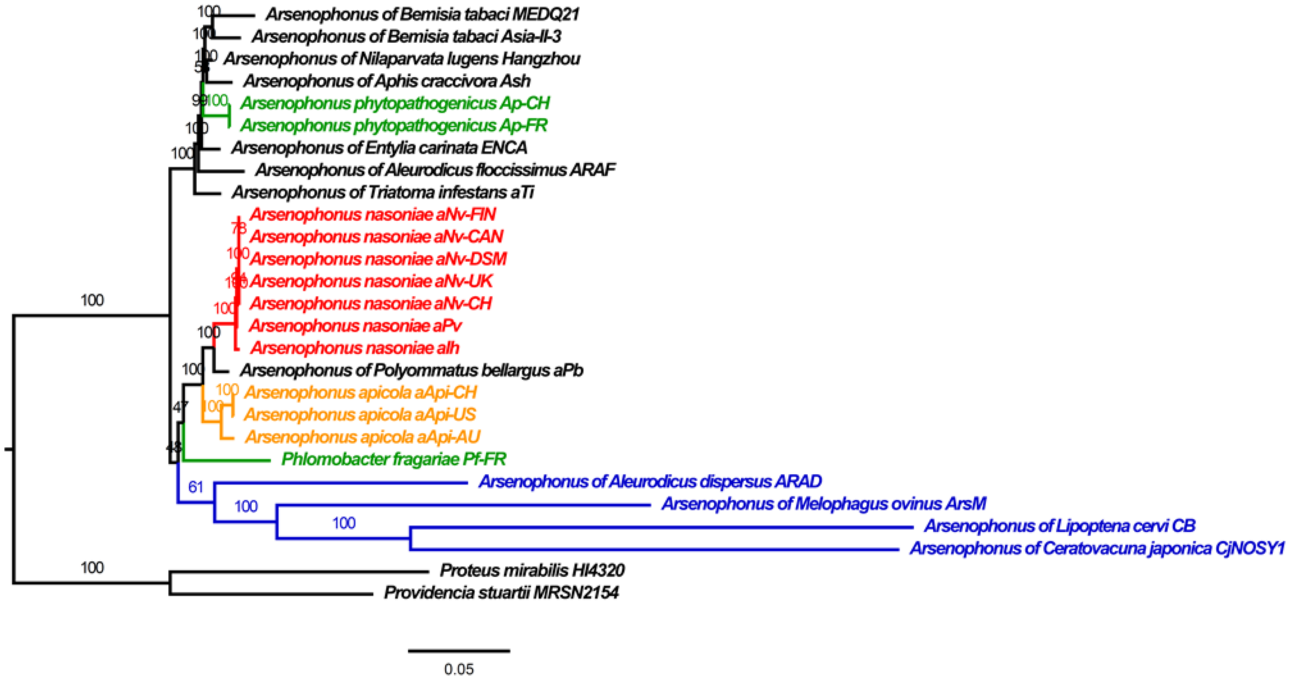

(B) MrBayes

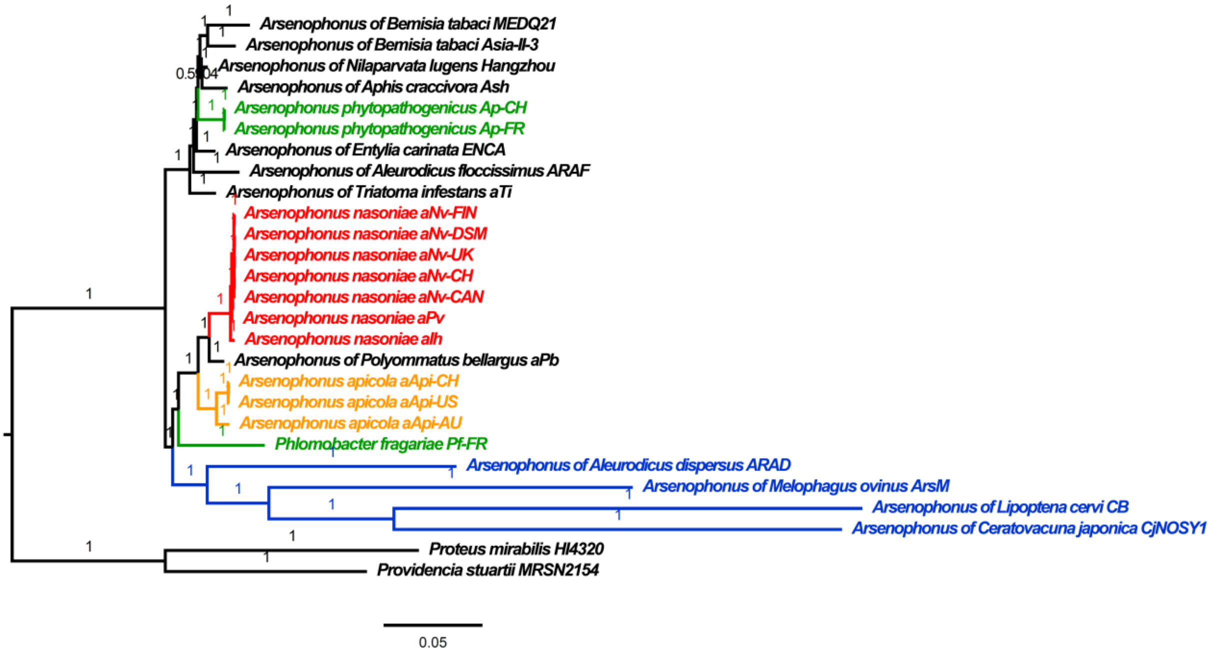

(C) PhyloBayes

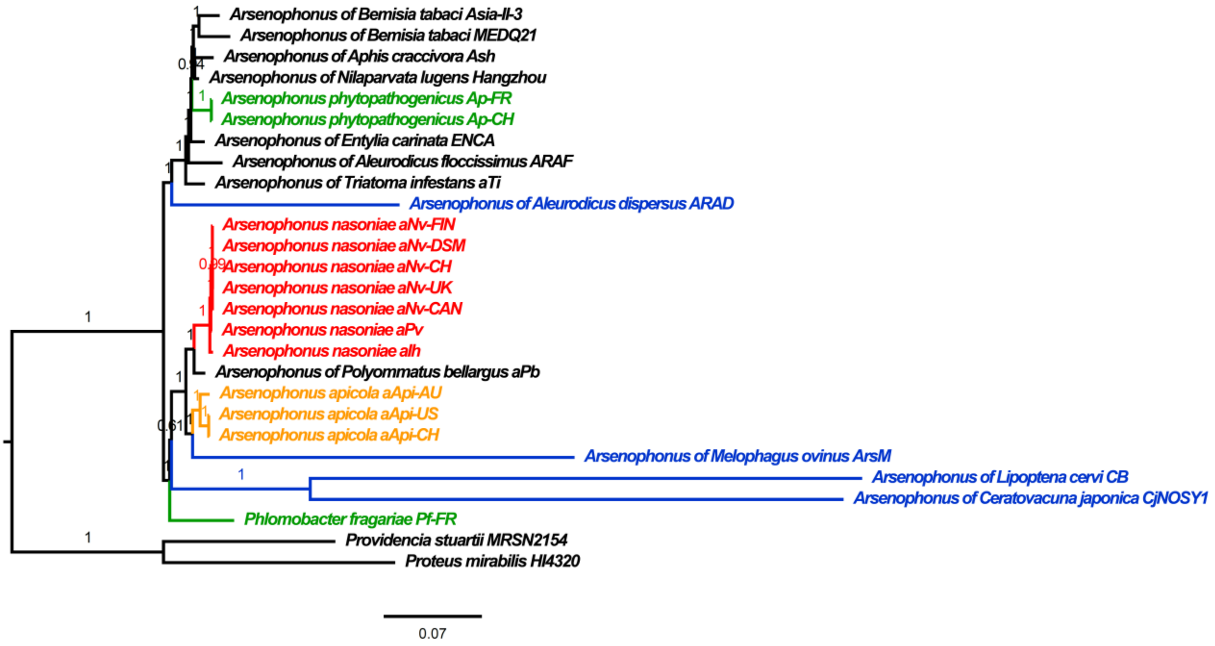

Figure S4. Phylogenomic analyses of *Arsenophonus* strains including the P-endosymbionts, based on 109 shared single copy protein-coding genes. *Providencia stuartii* and *Proteus mirabilis* were used to root the trees. (A) ML phylogenomic tree produced using IQtree. Branch support is based on 1000 bootstrap iterations. (B) Bayesian phylogenetic tree produced using MrBayes from two independent runs with four chains each, running for 3,000,000 generations. Posterior probabilities are indicated on each branch. (C) Bayesian phylogenetic tree produced using PhyloBayes from two independent chains run for 10,000 generations. Posterior probabilities are indicated on each branch.

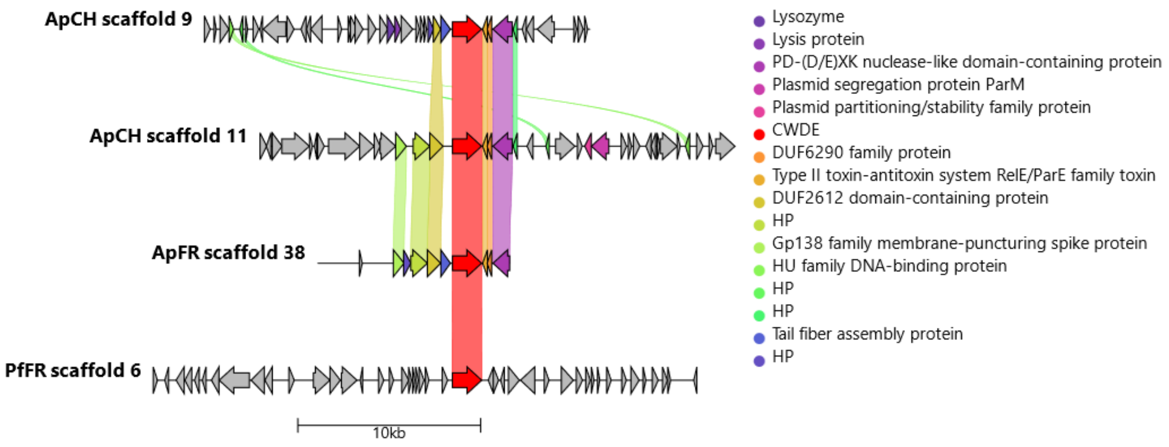

Figure S5. Gene synteny in the genomic context of CWDEs in Ap and Pf genomes.

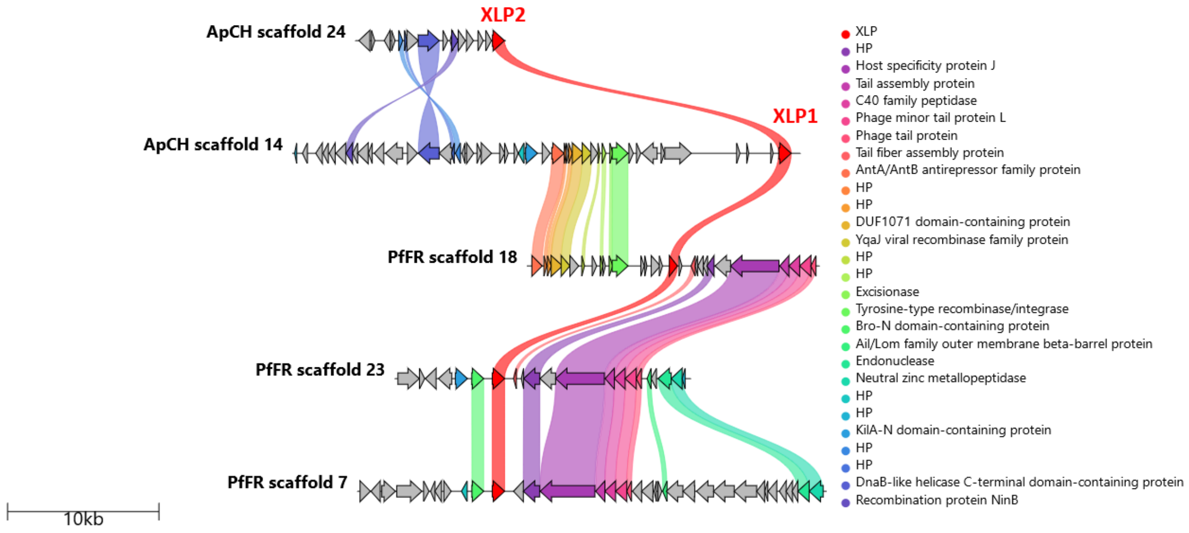

Figure S6. Gene synteny in the genomic context of XLPs in Ap and Pf genomes.

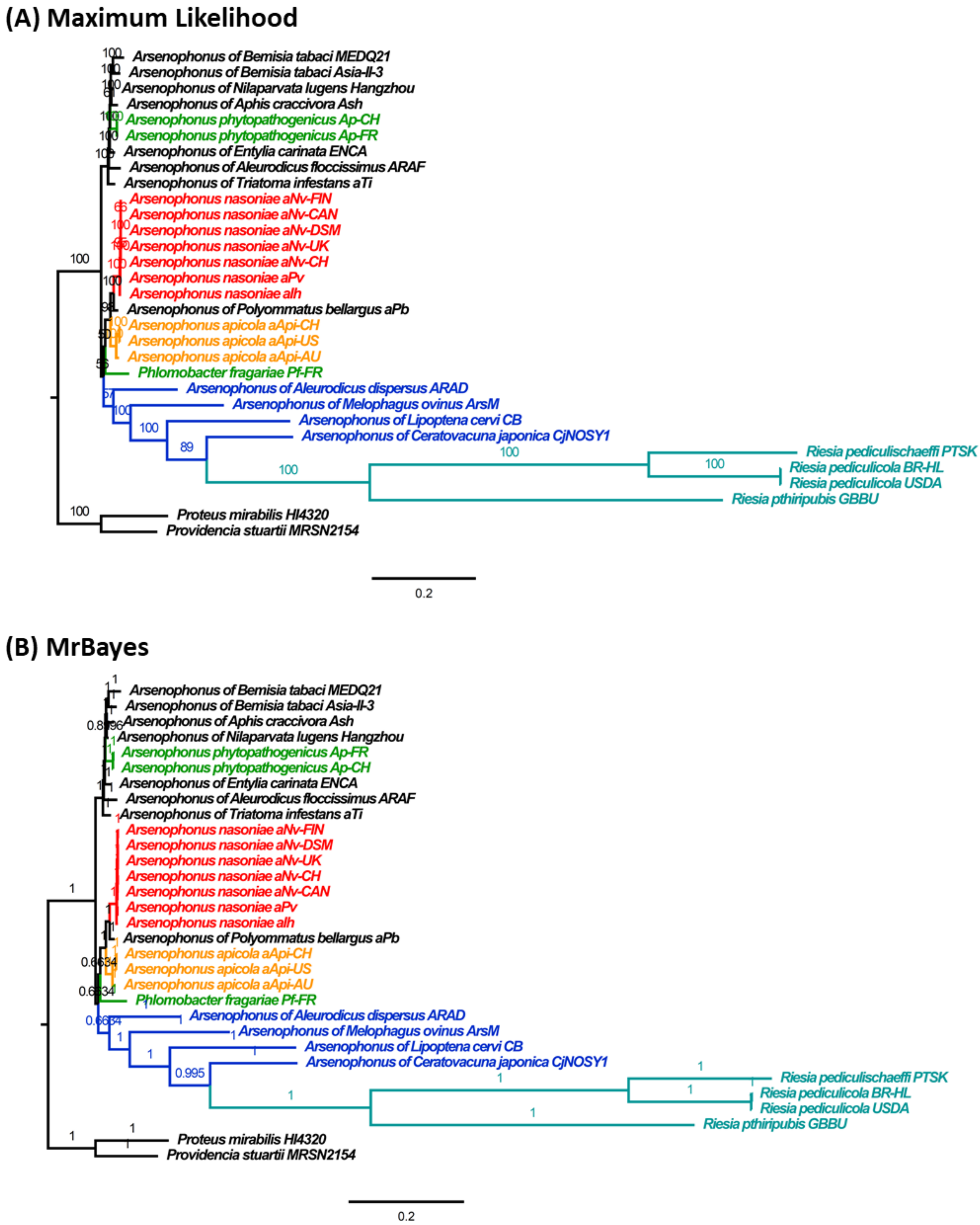

Figure S7. *Riesia* clusters together with the P-endosymbionts, forming extremely long branches. Phylogenomic analyses of *Arsenophonus* and *Riesia* spp., based on 74 shared single copy protein-coding genes. *Providencia stuartii* and *Proteus mirabilis* were used to root the trees. (A) ML phylogenomic tree produced using IQtree. Branch support is based on 1000 bootstrap iterations. (B) Bayesian phylogenetic tree produced using MrBayes from two independent runs with eight chains each, running for 3,000,000 generations. Posterior probabilities are indicated on each branch.

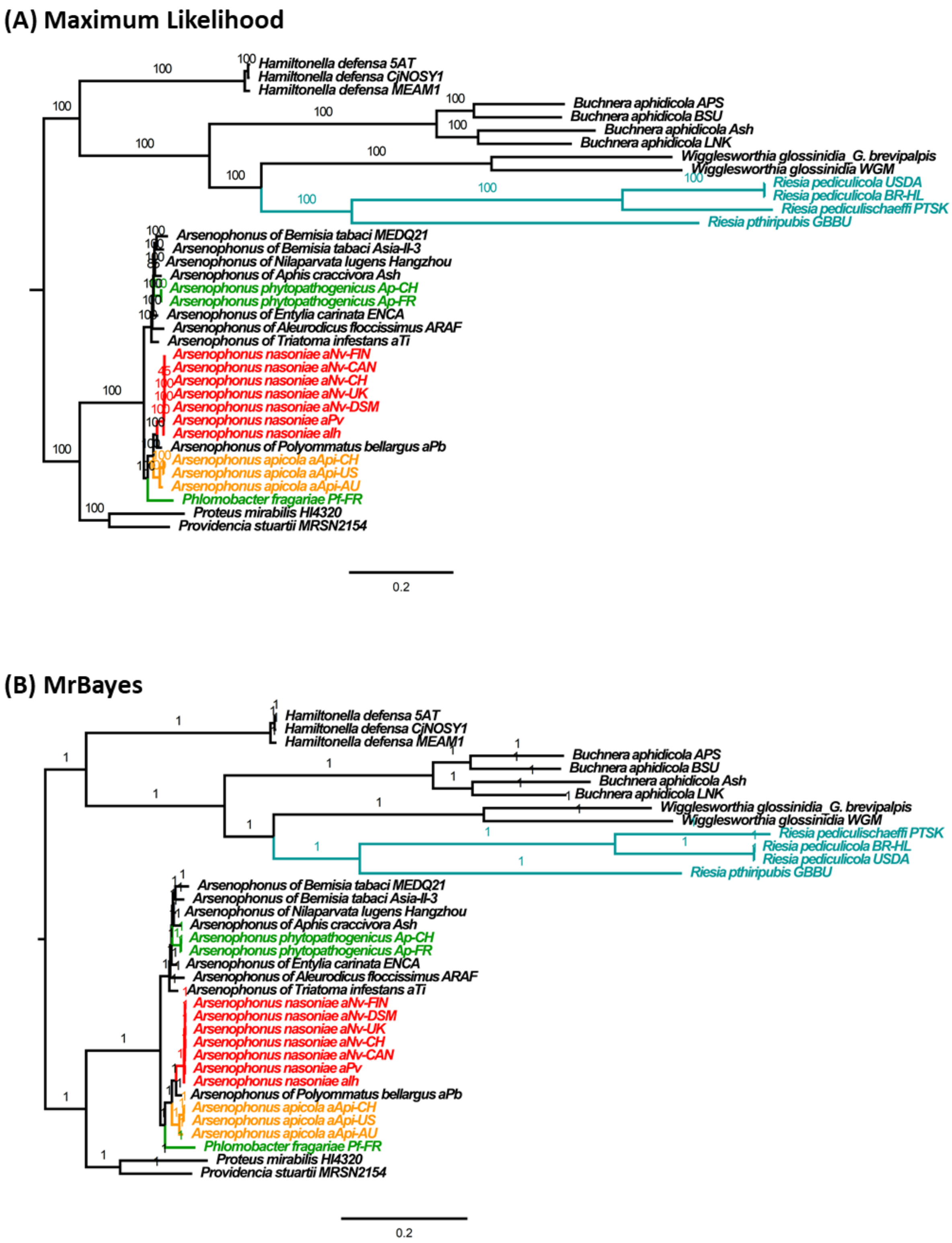

Figure S8. When removing the long-branched *Arsenophonus* strains and adding additional insect endosymbionts from the Enterobacteriaceae family, *Riesia* no longer clusters within the genus *Arsenophonus*. Phylogenomic analyses based on 73 shared single copy protein-coding genes. (A) ML phylogenomic tree produced using IQtree. Branch support is based on 1000 bootstrap iterations. (B) Bayesian phylogenetic tree produced using MrBayes from two independent runs with eight chains each, running for 3,000,000 generations. Posterior probabilities are indicated on each branch.

(A) Maximum Likelihood

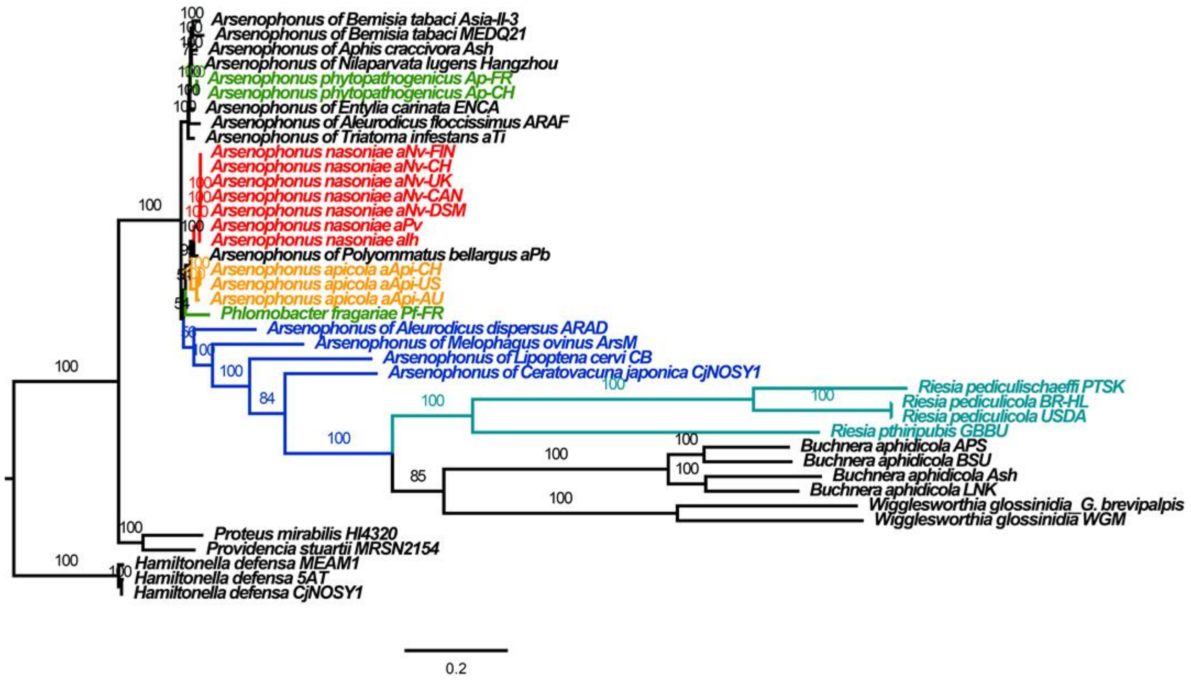

(B) MrBayes

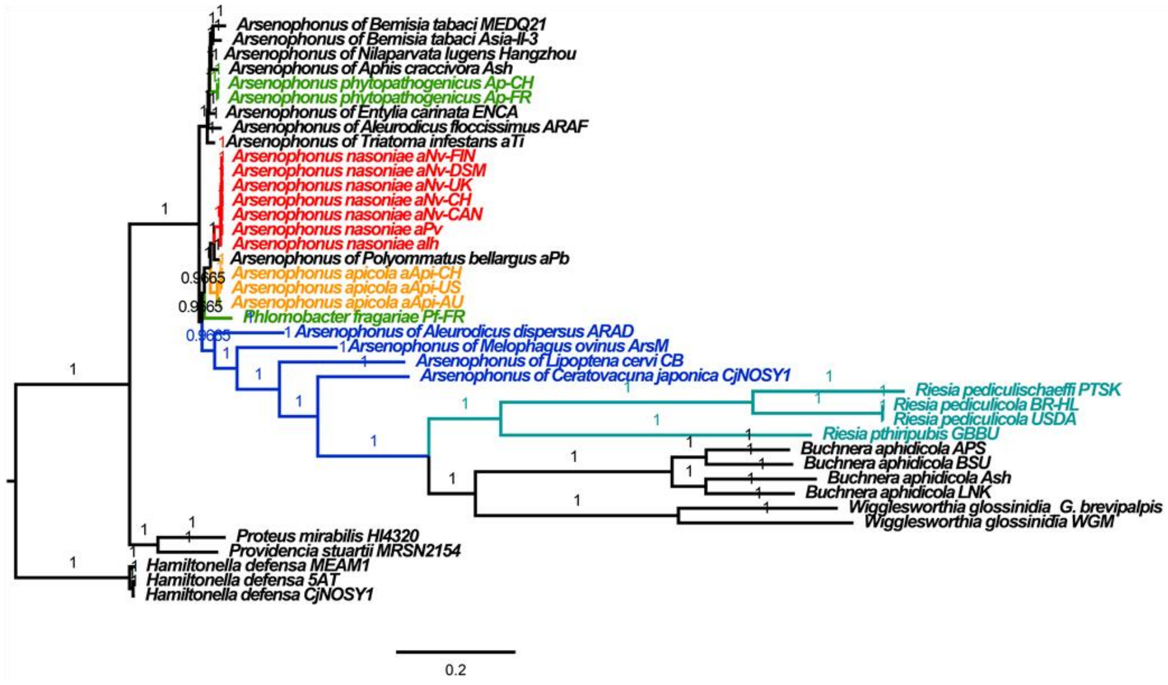

Figure S9. Adding the long-branched *Arsenophonus* strains to the same analysis, "pulls" not only *Riesia*, but also *Buchnera* and *Wigglesworthia* into the genus *Arsenophonus*. Phylogenomic analyses based on 55 shared single copy protein-coding genes. (A) ML phylogenomic tree produced using IQtree. Branch support is based on 1000 bootstrap iterations. (B) Bayesian phylogenetic tree produced using MrBayes from two independent runs with eight chains each, running for 3,000,000 generations. Posterior probabilities are indicated on each branch.
